# Long-term cross-variant Fc-mediated immune responses against SARS-CoV-2 induced by a heterologous adenoviral/inactivated virus prime-boost vaccination strategy

**DOI:** 10.1038/s41541-026-01483-z

**Published:** 2026-05-22

**Authors:** Doyoung Kim, Jung Hyuk Lee, Junhyeon Lee, Ju Yeon Park, Yuna Shin, Soo Ji Kim, BeomMin Cheon, Sumin Lee, Eunjin Cho, Deok Ryun Kim, Ryan P. McNamara, Cheol-Heui Yun, Mohamadou Siribie, Asma Binte Aziz, Birkneh Tilahun Tadesse, Florian Marks, Sue Kyoung Jo, Hyon Jin Jeon, Raphaël Rakotozandrindrainy, Ilesh V. Jani, Jae Seung Yang, Byoung-Shik Shim, Manki Song

**Affiliations:** 1 International Vaccine Institute, Seoul, Republic of Korea; 2 Department of Agricultural Biotechnology, and Research Institute of Agriculture and Life Sciences, Seoul National University, Seoul, Republic of Korea; 3 Department of Molecular Biosciences, The University of Texas at Austin, Austin, TX USA; 4 Department of Immunology and Infectious Diseases, Harvard T. H. Chan School of Public Health, Boston, MA USA; 5 Ragon Institute of Mass General Brigham, MIT, and Harvard, Cambridge, MA USA; 6 Institutes of Green Bio Science & Technology, Seoul National University, Pyeongchang, Republic of Korea; 7 Center for Food and Bioconvergence, Seoul National University, Seoul, Republic of Korea; 8 Madagascar Institute for Vaccine Research, University of Antananarivo, Antananarivo, Madagascar; 9 Instituto Nacional de Saúde, Maputo, Mozambique

**Keywords:** Immunology, Microbiology

## Abstract

Limited vaccine availability and logistical barriers during the COVID-19 pandemic have hindered homologous boosting in resource-limited regions. Therefore, heterologous prime-boost regimens have gained attention as versatile and practical alternatives. We evaluated the immunogenicity and longevity of four vaccine regimens in adults from Mozambique and Madagascar: single-dose Ad26.COV2.S (Ad26.S), homologous BBIBP-CorV (BBIBP), and two heterologous combinations (BBIBP-Ad26.S and Ad26.S-BBIBP, in prime-boost order). Using systems serology, we characterized Fc-mediated antibody responses against SARS-CoV-2 wild-type and Omicron BA.1. Among all regimens, Ad26.S-BBIBP elicited broad and sustained humoral immunity for up to 6 months, with enhanced IgG levels, Fcγ receptor binding, and Fc-mediated effector functions. These responses declined more slowly than with single-Ad26.S. Compared to the other regimens, antibody-dependent natural killer cell activation (ADNKA) emerged as a distinct characteristic of durable cross-variant immunity in Ad26.S-BBIBP recipients. These findings highlight that the Ad26.S-BBIBP regimen is immunologically advantageous, with Fc-mediated effector functions, particularly ADNKA, representing an important characteristic of its relatively durable and cross-strain protection even as neutralizing antibody titers decline.

## Introduction

The COVID-19 pandemic has highlighted profound global health disparities, particularly in terms of vaccine access and distribution between low- and middle-income versus high-income countries^1,2^. Limited vaccine supplies, logistical hurdles, and insufficient infrastructure have hindered timely and widespread vaccination, especially in African countries, consequently prolonging the pandemic and increasing case numbers and mortality^3–5^. These constraints have also made homologous boosting (using the same vaccine for both priming and boosting) impractical in some regions^6,7^, revealing the urgent need for more flexible and pragmatic prime-boost vaccination strategies, particularly in the face of emerging variants and waning immunity. Heterologous prime-boost regimens, which involve administration of different vaccine platforms encoding the same antigen, have emerged as promising alternatives^7,8^. This approach takes advantage of the distinct immune activation pathways triggered by different vaccine platforms, with the priming and boosting sequence playing a pivotal role in shaping the magnitude, quality, and durability of the immune response^9^. This strategy offers opportunities for enhanced immunogenicity, improved variant coverage, greater logistical flexibility, and a wider range of boosting options^10–15^.

Among currently authorized COVID-19 vaccines, Ad26.S (Johnson & Johnson) and BBIBP (Sinopharm) are some of the most widely deployed in sub-Saharan Africa^16^. Ad26.S, a viral vector vaccine encoding the full-length spike (S) protein of severe acute respiratory syndrome coronavirus-2 (SARS-CoV-2) in a prefusion-stabilized form, induces strong antibody and T-cell responses and durable immunity, even following a single low-dose injection^17,18^. In contrast, BBIBP contains inactivated SARS-CoV-2 virus particles with aluminum hydroxide adjuvants and generates safe and robust antibody responses with two doses administered at a 21–28-day interval^19–22^. Although antibody profiles were elicited to the target S, breadth to antigenically diverged S, such as those of Omicron variants of concern (VOCs), was reduced^23–25^. A previous study suggested that viral vector boosters following a primary inactivated vaccine series could improve binding antibody and cross-variant neutralizing antibody (NAb) levels^26,27^. However, the comprehensive immunological profile of Ad26.S and BBIBP in heterologous combinations, particularly the impact of the prime-boost sequence, immunogenicity, long-term durability, and cross-variant protection, remains poorly characterized.

In evaluating COVID-19 vaccine efficacy, NAb titers have served as the primary metric and were strongly correlated with protection^28^. However, as VOCs emerged, the predictive power of NAb levels has decreased^29^. Interestingly, protection against severe disease and mortality often persists even as NAb titers decline^30^, suggesting that additional immune mechanisms contribute to sustained protection. The Fc-mediated non-neutralizing antibody (nNAb) is involved in functions such as antibody-dependent cellular phagocytosis, complement deposition, and natural killer (NK) cell responses, and has gained increased attention as key components of durable immune responses to SARS-CoV-2^30,31^. These functions can be extensively characterized using systems serology, a high-dimensional profiling platform that reveals the breadth and quality of Fc–effector activity, thereby providing a more holistic understanding of humoral immunity^32^. Given the importance of understanding these responses amid evolving variants, it remains unclear whether heterologous regimens of Ad26.S and BBIBP can elicit and boost these nNAb-mediated immune functions.

In this study, we comprehensively evaluated the immunogenicity and durability of Ad26.S and BBIBP vaccines administered in both homologous and heterologous prime-boost regimens using a systems serology approach to characterize Fc-mediated functional humoral responses across SARS-CoV-2 variants. Our results demonstrated that Ad26.S and BBIBP in prime-boost order induces robust and sustained Fc-mediated effector functions, particularly durable antibody-dependent NK cell activation (ADNKA). Correspondingly, ADNKA-activating features such as IgG, IgG1, IgG3, and FcγR3A were uniquely enriched in this heterologous regimen. These antibody features and functions persisted for up to 6 months post-priming and were effective against both wild-type (WT) SARS-CoV-2 and the Omicron BA.1 variant. Our findings highlight the immunological advantages of this specific heterologous regimen and its potential to inform strategic vaccination programs for durable cross-variant protection, particularly in low- and middle-income countries.

## Results

### Different prime-boost vaccine regimens induce distinct humoral immune profiles

**Fig. 1:**
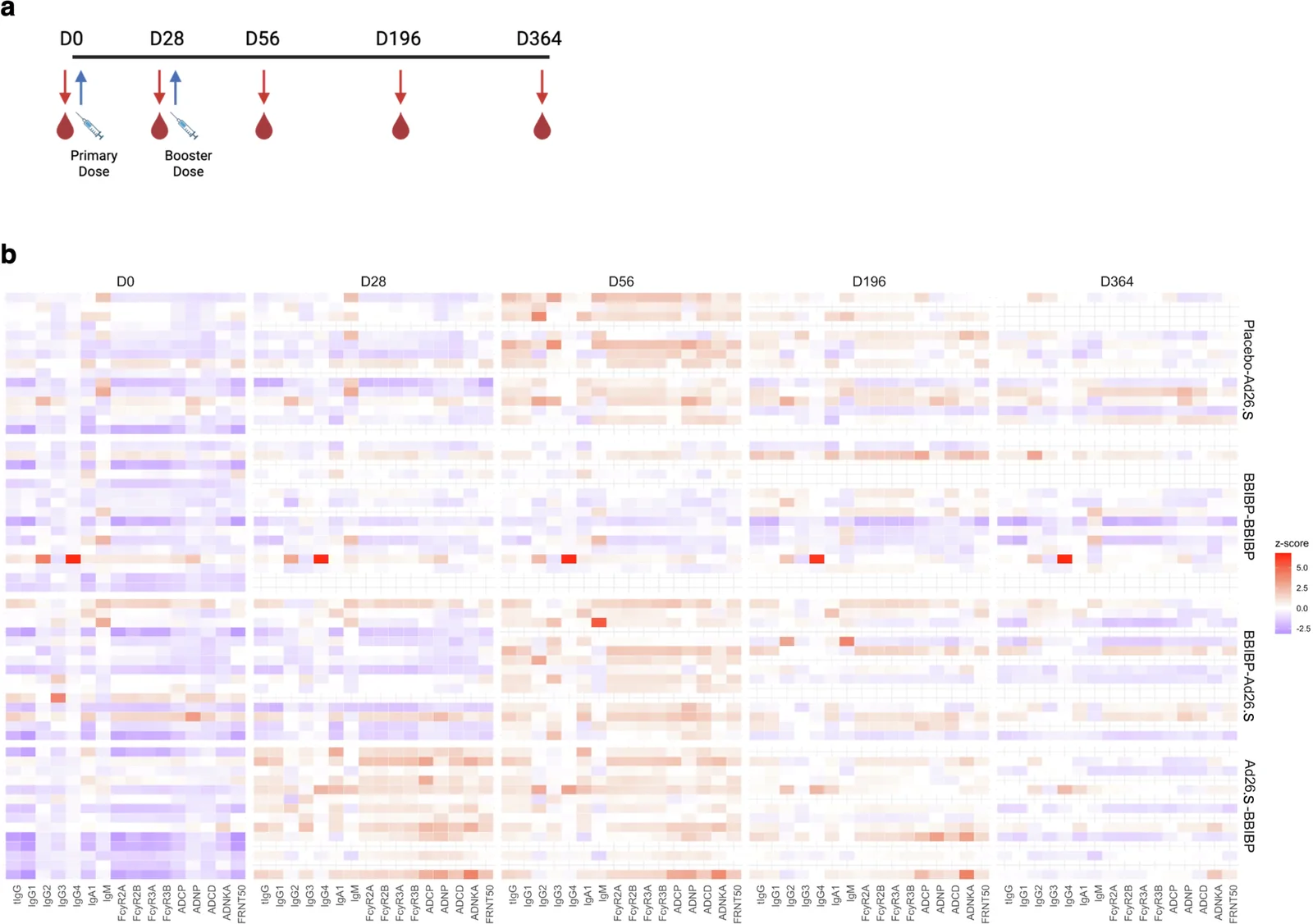
Heterogeneity of immune responses across prime-boost regimens against SARS-CoV-2 WT. **a** Schematic of the vaccination schedule and sample collection timeline. Participants were randomly assigned to one of four vaccine regimen groups: Placebo-Ad26.S (*n* = 15), BBIBP-BBIBP (*n* = 16), BBIBP-Ad26.S (*n* = 15), and Ad26.S-BBIBP (*n* = 14). Vaccinations with BBIBP and Ad26.S were administered on D0 and D28 according to the respective prime-boost combinations. Sera were collected at multiple time points up to D364 and analyzed using systems serology. The panel was created with Biorender. **b** Heatmap depicting humoral immune responses against SARS-CoV-2 WT S protein across the four vaccine groups. Rows represent individual participants by groups, whereas columns correspond to immune features (z-scored), including isotype, subclass, FcγR-binding, antibody effector functions, and neutralization, separated by each time point. High responses are depicted in red, and low responses are shown in blue. Empty cells represent missing samples either due to participant absence at sample collection time points or exclusion of suspected breakthrough infection samples identified using the interquartile range (IQR) 1.5× rule.

Serum samples from a Phase II observer-blinded randomized clinical trial (NCT04998240)^33^ were analyzed to evaluate the serological responses induced by different prime-boost vaccine regimens involving Ad26.S and BBIBP. The study enrolled healthy adults aged 18–65 years from Mozambique and Madagascar with no prior history of COVID-19 vaccination. Participants were recruited between December 2021 and September 2022 during the Omicron BA.1 outbreak^34,35^. Due to the rapid spread of the virus, the majority of the populations in the two countries had already been exposed to the Omicron VOC at the time of recruitment. This limited the ability to enroll SARS-CoV-2-naive individuals; therefore, the study cohort included both seropositive and seronegative participants. Participants were randomly assigned to one of four vaccine regimen groups: placebo followed by a single Ad26.S dose (Placebo-Ad26.S), homologous BBIBP prime-boost (BBIBP-BBIBP), heterologous BBIBP prime followed by Ad26.S boost (BBIBP-Ad26.S), and heterologous Ad26.S prime followed by BBIBP boost (Ad26.S-BBIBP). A subset of 60 participants (14–16 in each group) was randomly selected from the main cohort. The prime dose was administered on day 0 (D0), followed by a booster dose on day 28 (D28). Serum samples were collected on days 0, 28, 56, 196, and 364 (Fig. 1a). A summary of the final sample set for each vaccine regimen is presented in Table 1.

**Table 1:** Study design and vaccine regimen details for each arm in the immunology subset

Vaccination strategy	Primer dose (D0)	Booster dose (D28)	Number of participants				
			D0	D28	D56	D196	D364
Standard vaccination	Placebo	Ad26.COV2.S	15	12	12	11	10
	BBIBP-CorV	BBIBP-CorV	16	11	12	11	10
Heterologous vaccination	BBIBP-CorV	Ad26.COV2.S	15	14	13	11	10
	Ad26.COV2.S	BBIBP-CorV	14	12	12	10	8

Participants were divided into four groups based on the vaccination approaches—Placebo-Ad26.S, BBIBP-BBIBP, BBIBP-Ad26.S, and Ad26.S-BBIBP—reflecting either standard or heterologous regimens. The number of participants at each time point is shown after excluding samples that exceeded the upper NP-IgG threshold used to identify and remove suspected breakthrough infections.

To characterize the SARS-CoV-2-specific humoral immune response in the four vaccination groups, we employed systems serology to comprehensively profile peak and long-term responses to the SARS-CoV-2 WT S protein, the target of vaccination. Analyses included measurements of antibody isotypes and subclasses, FcγR binding, and Fc-mediated effector functions to SARS-CoV-2 S variants, including antibody-dependent complement deposition, cellular phagocytosis, neutrophil phagocytosis (ADNP), and natural killer cell activation (ADNKA) (Supplementary Table 1). We assessed the overall immune response using Z-scores, which reflect coordinated deviations from baseline across multiple immune features and vaccination groups. Distinct immunological profiles were observed among the four vaccine regimen groups (Fig. 1b). In the Placebo-Ad26.S group, a single dose of Ad26.S induced a robust and durable immune response, as indicated by higher Z-scores (red) compared with baseline levels and responses following BBIBP, which were represented by lower Z-scores (blue). They peaked on day 56 (D56) and were moderately sustained through day 196 (D196). In contrast, the homologous BBIBP-BBIBP regimen produced only modest changes in responses over time. In the heterologous BBIBP-Ad26.S group, immune responses were initially minimal but improved following Ad26.S boost in terms of both magnitude and durability; however, overall immunogenicity remained largely comparable to that observed with a single dose of Ad26.S. Notably, the Ad26.S-BBIBP regimen elicited a strong early immune response, and many features appeared to persist up to D196.

### Ad26.S-BBIBP heterologous prime-boost regimen induces sustained longitudinal antibody levels

**Fig. 2:**
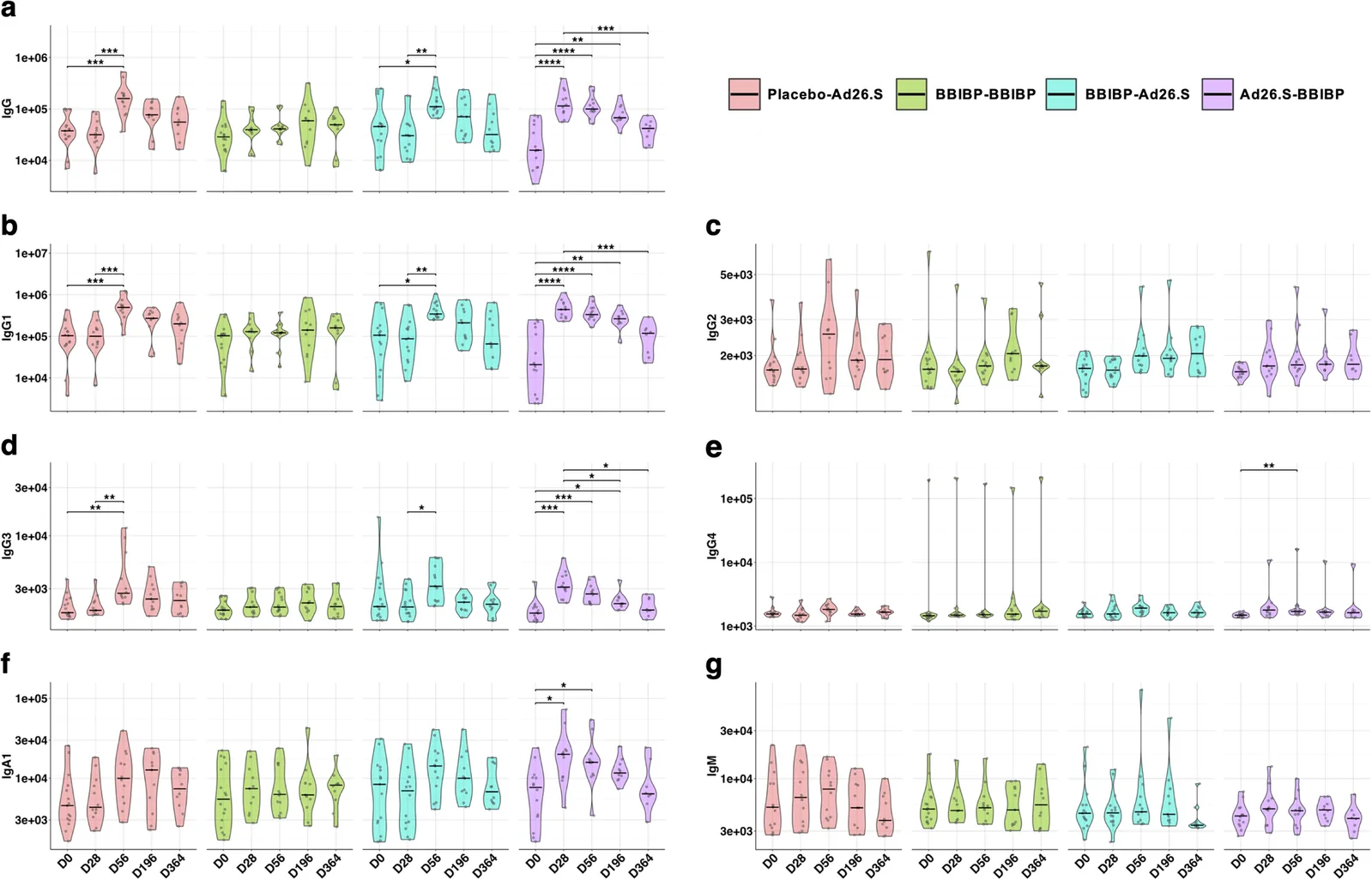
Longitudinal antibody profiles against SARS-CoV-2 WT S antigen across vaccine regimens. Violin plots show the longitudinal dynamics of SARS-CoV-2 WT S-specific antibody responses at five time points: baseline (D0; prior to vaccination), post-primary and booster doses (D28 and D56), and extended follow-up (D196 and D364). The vaccine groups are displayed from left to right as follows: Placebo-Ad26.S, BBIBP-BBIBP, BBIBP-Ad26.S, and Ad26.S-BBIBP. Individual data points are shown in gray, with medians indicated by a black dashed line. The y-axes represent the geometric mean fluorescence intensity (GMFI) of **a** IgG, **b** IgG1, **c** IgG2, **d** IgG3, **e** IgG4, **f** IgA1, and **g** IgM. All assays were performed in technical quadruplicate. Statistical comparisons between time points within each group were conducted using Wilcoxon signed-rank test with Bonferroni correction (*P < 0.05, **P < 0.01, ***P < 0.001, and ****P < 0.0001). Statistical significance was annotated solely for time points compared to D0 and D28.

To deeply assess the peak and waning dynamics of vaccine-induced humoral immunity following different vaccine regimens, we conducted univariate analyses of SARS-CoV-2 WT S-specific antibody isotypes and subclasses within each group. The homologous BBIBP-BBIBP group exhibited minimal changes over time, with no considerable increase in antibody levels across all isotypes and subclasses following either dose (Fig. 2; green), suggesting limited antibody responses to the BBIBP vaccine in this cohort. In contrast, Ad26.S vaccination significantly elevated SARS-CoV-2 WT S-specific IgG, IgG1, and IgG3 responses, resulting in peak immunogenicity in the Placebo-Ad26.S and BBIBP-Ad26.S groups on D56 and Ad26.S-BBIBP group on D28 (Fig. 2a, b, d; red, turquoise, and purple). However, neither those who received a single shot of Ad26.S nor those who were primed with BBIBP before the Ad26.S boost induced sustained antibody responses, as all features returned to statistically indistinguishable levels at D196 compared to D0 (baseline). This result indicates that BBIBP, as a primer prior to Ad26.S boosting, has a limited immunological advantage. In contrast, in Ad26.S-primed individuals boosted with BBIBP, antibody levels were significantly elevated through D196 compared to those at baseline (D0) (*P* = 0.009 for IgG, *P* = 0.004 for IgG1, and *P* = 0.024 for IgG3), with only a modest decline by day 364 (D364), at which point the median titers remained statistically above baseline levels for several features. Other antibody isotypes and subclasses, including IgG2, IgG4, and IgA1 showed minimal or no changes across all vaccine regimens. Also, IgM showed no statistical elevation over D0 for any group, indicating that most phenotypes observed were likely not de novo (Fig. 2c, e–g). We extended our analysis to include SARS-CoV-2 WT S1, S2, and receptor-binding domain (RBD) antigens to explore whether antibody responses differed across specific regions of the S protein. Consistent with the patterns observed for full-length S, antibody responses to all three subdomains exhibited similar peaks and waning kinetics across all vaccination groups, with considerably sustained levels of IgG, IgG1, and IgG3 by D196 and D364 in the Ad26.S-BBIBP group (Supplementary Figs. 1–3). These findings support that BBIBP boosting after Ad26.S priming extends the durability of IgG, IgG1, and IgG3 antibody levels.

### FcγR-binding responses track with antibody kinetics across vaccine regimens

**Fig. 3:**
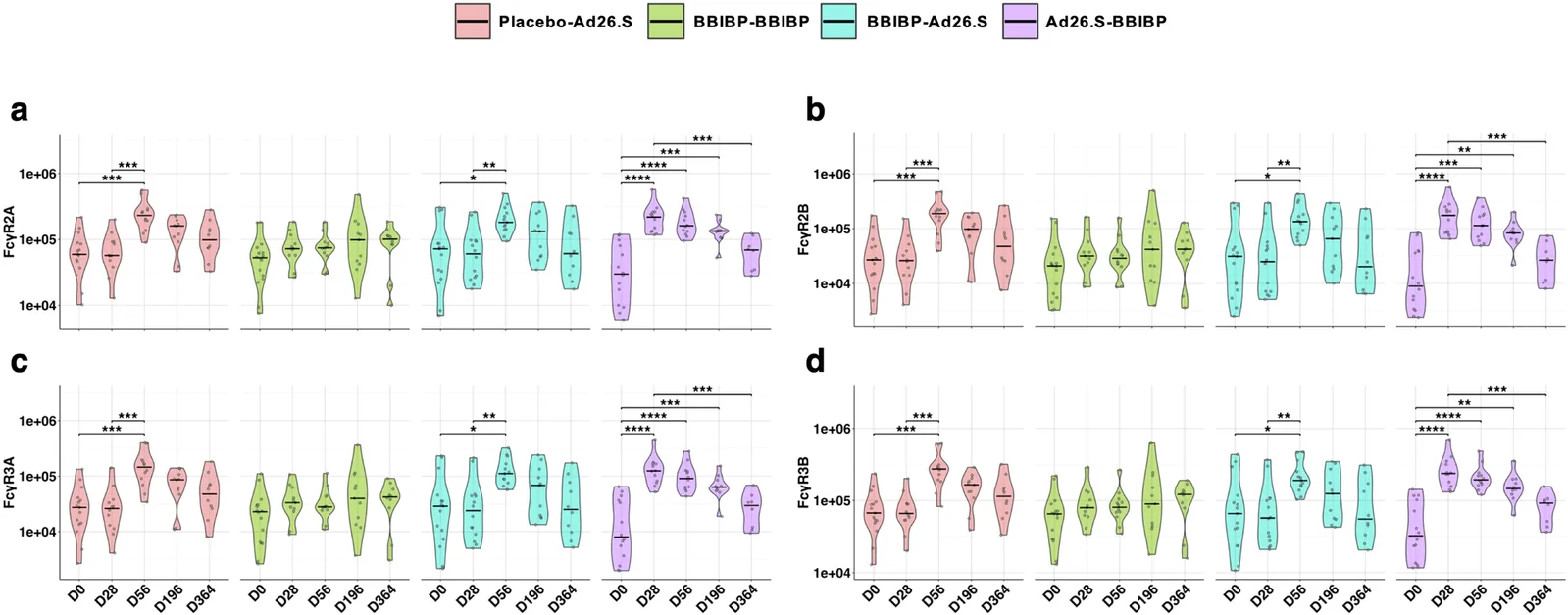
FcγR-binding antibody responses to SARS-CoV-2 WT S across vaccine regimens over time. Violin plots present longitudinal FcγR binding levels against SARS-CoV-2 WT S protein up to D364 across four vaccine groups. The gray dots indicate individual measurements with black dashed lines marking medians. The y-axes show GMFI of **a** FcγR2A-, **b** FcγR2B-, **c** FcγR3A-, and **d** FcγR3B-binding antibodies. All assays were performed in technical quadruplicate. Time point comparisons within groups were analyzed using Wilcoxon signed-rank tests adjusted with Bonferroni correction (*P < 0.05, **P < 0.01, ***P < 0.001, and ****P < 0.0001). Statistical annotations are provided exclusively for comparison between D0 and D28.

It is well-established that antibodies mediate effector functions by engaging with Fc receptors expressed on innate immune cells^36^. Thus, to determine whether FcγR-binding follow similar kinetics to overall antibody titers, we evaluated the binding of SARS-CoV-2 WT S-specific antibody to the four low-affinity human Fcγ receptors (FcγR2A, FcγR2B, FcγR3A, and FcγR3B) across vaccine regimen groups. We observed that FcγR-binding dynamics closely followed those of the overall antibody responses, with comparable peak and waning patterns across all four receptors (Fig. 3). Ad26.S administration markedly augmented FcγR-binding activity (red, turquoise, and purple), whereas the homologous BBIBP-BBIBP regimen elicited only modest responses (green). In both the Placebo-Ad26.S and BBIBP-Ad26.S groups, FcγR-binding responses peaked at D56 and declined thereafter, with limited and insignificant persistence through D196 compared to D0. In contrast, the Ad26.S-BBIBP group consistently displayed the most durable FcγR-binding responses across all four FcγRs, remaining significantly elevated up to D196 relative to baseline (*P* = 0.001 for FcγR2A, *P* = 0.004 for FcγR2B, *P* = 0.001 for FcγR3A, and *P* = 0.002 for FcγR3B). A similar trend was observed when FcγR-binding was measured against the S1, S2, and RBD domains, which tracked closely with full-length S-specific binding, as well as the isotype and subclass patterns (Supplementary Figs. 4–6). Collectively, these findings indicate that FcγR-binding profiles closely align with those of antibody isotypes and subclasses, supporting the expectation of sustained Fc-mediated effector functions in Ad26.S-BBIBP regimens.

### Ad26.S-BBIBP regimen promotes robust and durable Fc-mediated effector functions and NAbs

**Fig. 4:**
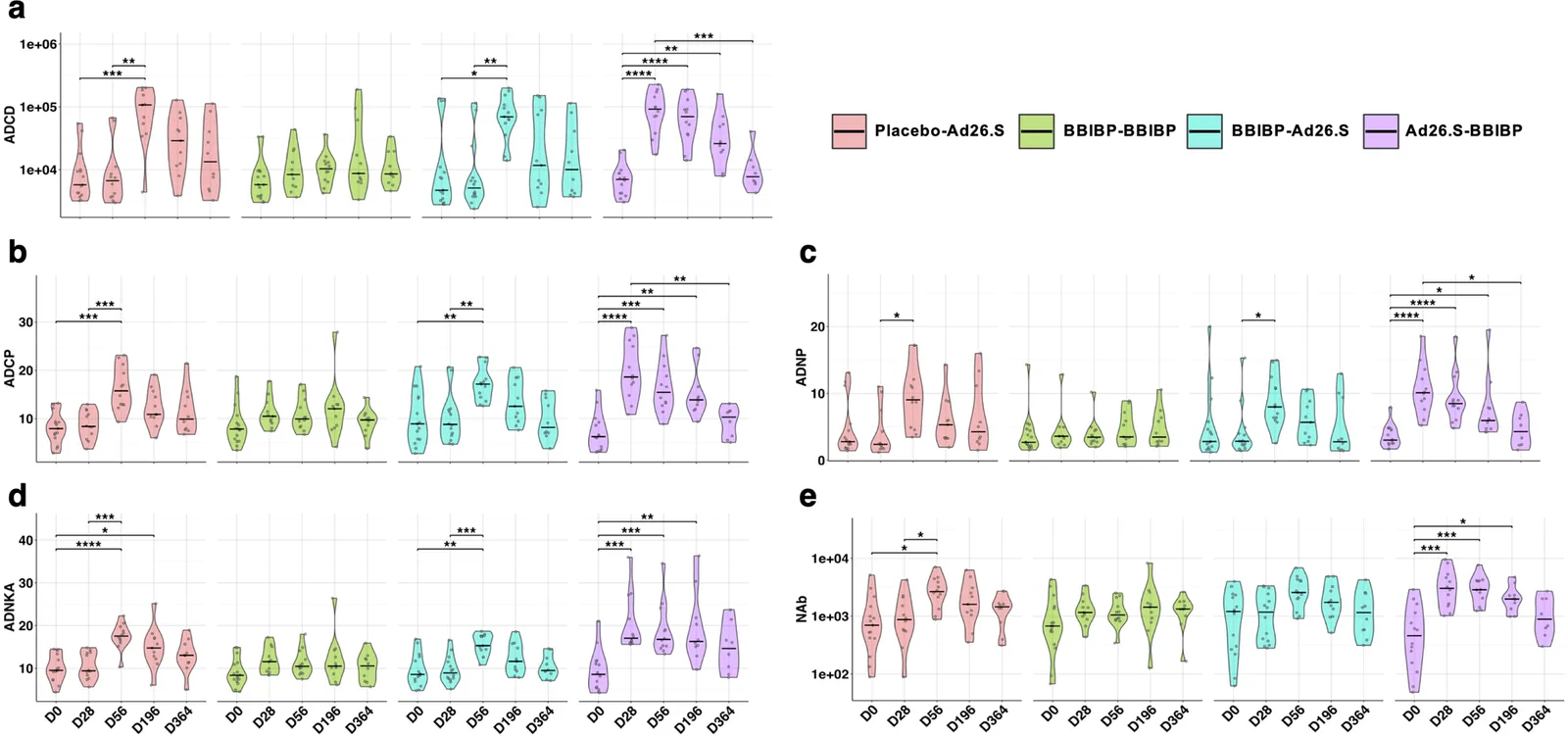
Fc-mediated functional antibody activity against SARS-CoV-2 WT S over time. Violin plots showing Fc-mediated effector functions and neutralization of the SARS-CoV-2 WT S protein measured up to D364 in the four vaccine groups. Functional assays included **a** antibody-dependent complement deposition (ADCD; C3 deposition GMFI), **b** cellular phagocytosis (ADCP; phagoscores), **c** neutrophil phagocytosis (ADNP; phagoscores), and **d** natural killer cell activation (ADNKA; %CD107a+ cells). **e** Neutralization was determined using the Focus Reduction Neutralization Test (FRNT50) titer. Individual values are shown as gray dots, and medians are marked with black dashed lines. The assays were performed in quadruplicate. Paired comparisons within groups over time were conducted using Wilcoxon signed-rank test with Bonferroni correction (*P < 0.05, **P < 0.01, ***P < 0.001, and ****P < 0.0001). Significant markers are provided only for comparisons relative to D0 and D28.

Building upon the FcγR-binding results, we next assessed SARS-CoV-2 WT S-specific Fc-mediated effector functions, including ADCD, ADCP, ADNP, and ADNKA, to further characterize longitudinal functional immune activity across vaccine regimens. Overall, the functional response dynamics showed similar patterns as those observed for antibody titers and FcγR binding (Fig. 4a–d). Ad26.S vaccination induced robust Fc-mediated functions (red, turquoise, and purple), whereas the homologous BBIBP-BBIBP regimen resulted in weaker responses (green). Consistently, the Ad26.S-BBIBP group exhibited the most durable functional responses, with all four effector functions remaining significantly elevated through D196 compared to those at baseline (*P* = 0.003 for ADCD, *P* = 0.003 for ADCP, *P* = 0.031 for ADNP, and *P* = 0.009 for ADNKA). Although the Placebo-Ad26.S and BBIBP-Ad26.S groups demonstrated similar peak-and-decline trajectories, a single dose of Ad26.S still led to significantly elevated NK cell degranulation by D196 (*P* = 0.045, Fig. 4d; red).

Moreover, neutralization was measured using the Focus Reduction Neutralization Test (FRNT) to assess the neutralizing ability of antibodies within each vaccine regimen. Neutralizing responses followed similar trends as Fc-mediated antibody responses (Fig. 4e). A single dose of Ad26.S significantly increased the peak neutralization titers on D56 (*P* = 0.017), whereas BBIBP-Ad26.S showed a similar but non-significant increase following the Ad26.S boost. Among all regimens, only the Ad26.S-BBIBP group showed significantly increased neutralizing activity from baseline to D196 (*P* = 0.015). Consistent with these findings, the single-dose Ad26.S and heterologous regimens exhibited higher responses than the BBIBP-BBIBP homologous regimen in WT-specific antibody levels, Fc receptor-binding and Fc-mediated effector functions following the booster dose, although no significant differences were observed among the groups after D56 (Supplementary Fig. 7). Together, these findings indicate that the Ad26.S-BBIBP regimen not only induces strong and durable functional nNAbs but also supports the long-term maintenance of NAb responses.

### Ad26.S-BBIBP vaccine regimen broadens and sustains functional humoral immune responses against Omicron BA.1

**Fig. 5:**
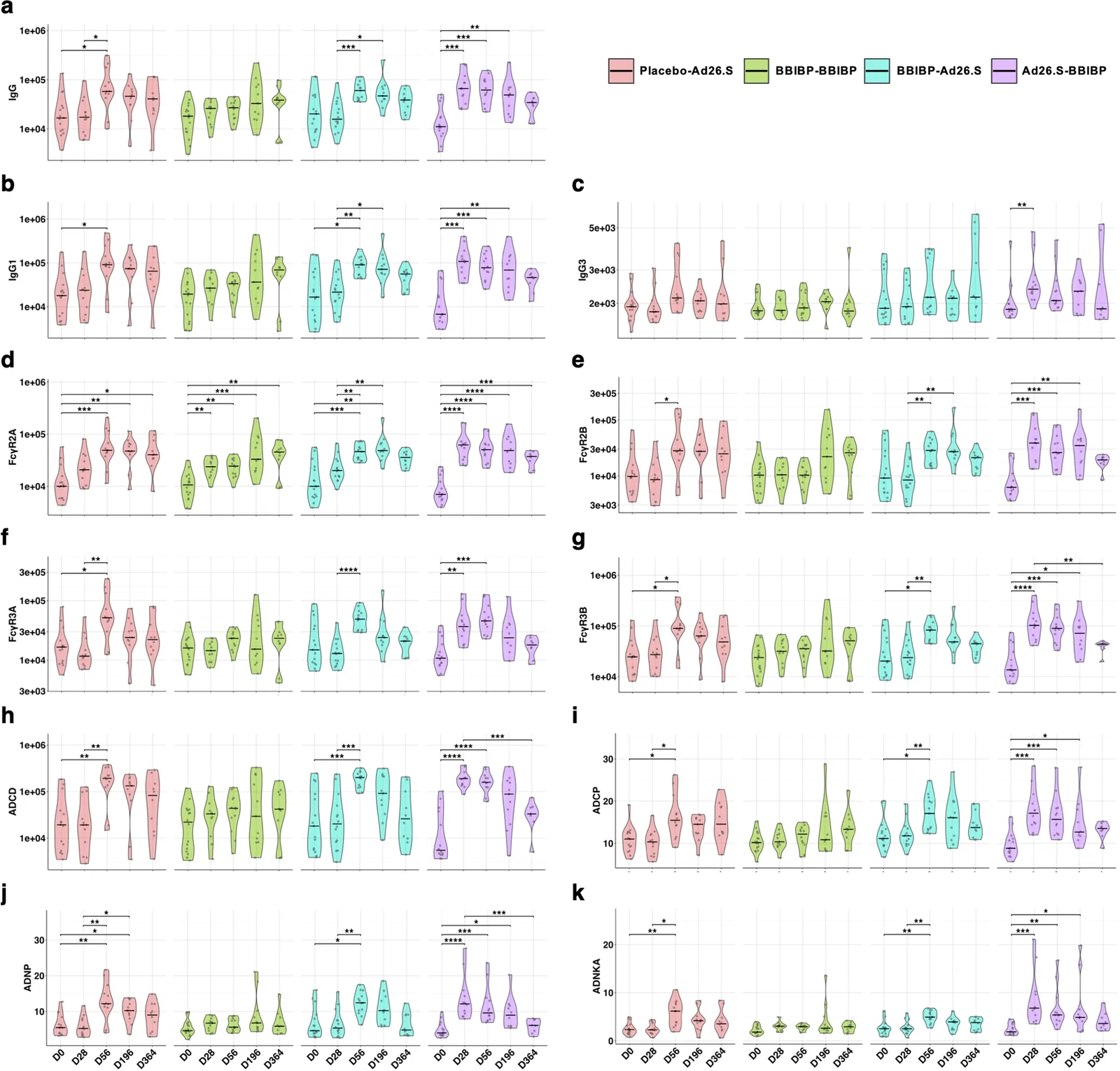
Antibody, FcγR binding, and functional profiles against SARS-CoV-2 Omicron BA.1 S across vaccine regimens over time. Violin plots illustrate antibody titers, FcγR binding, and functional responses against Omicron BA.1 S antigen at five time points. **a**–**c** show the GMFI of **a** IgG, **b** IgG1, and **c** IgG3. **d**–**g** display FcγR binding levels for **d** FcγR2A, **e** FcγR2B, **f** FcγR3A, and **g** FcγR3B. **h**–**k** show the functional activities measured via **h** antibody-dependent complement deposition (ADCD; C3 deposition GMFI), **i** cellular phagocytosis (ADCP; phagoscores), **j** neutrophil phagocytosis (ADNP; phagoscores), and **k** natural killer cell activation (ADNKA; %CD107a+ cells). All assays were performed in quadruplicate. Statistical analyses between time points within groups utilized Wilcoxon signed-rank tests with Bonferroni correction (*P < 0.05, **P < 0.01, ***P < 0.001, and ****P < 0.0001). Statistical significance is indicated only for comparisons between D0 and D28.

To further investigate the breadth of vaccine-induced immunity, we longitudinally analyzed Omicron BA.1 S-specific antibody isotypes and subclasses, FcγR binding, and Fc-mediated effector functions across all vaccination groups (Fig. 5; Supplementary Fig. 8; Supplementary Table 2). Consistent with the WT S-specific responses, IgG and IgG1 levels against BA.1 S significantly increased from baseline following Ad26.S vaccination in the Placebo-Ad26.S, BBIBP-Ad26.S, and Ad26.S-BBIBP groups (red, *P* = 0.044 and *P* = 0.037; turquoise, *P* = 0.037 for IgG1; purple, *P* = 0.0003 and *P* = 0.0004) (Fig. 5a, b). These antibody titers peaked at D56 in the Placebo-Ad26.S and BBIBP-Ad26.S groups but subsequently waned, showing no significant persistence. In contrast, although the homologous BBIBP-BBIBP group induced minimal responses, when BBIBP was used as a booster following Ad26.S priming, BA.1 S-specific IgG and IgG1 levels remained significantly elevated through D196 compared to those at the baseline (*P* = 0.009 and *P* = 0.007, respectively), suggesting enhanced durability against both the WT strain and BA.1. IgG3 responses against BA.1 S remained largely unchanged across groups, except for a transient increase on D28 in the Ad26.S-BBIBP group (Fig. 5c). This is not unexpected given the short half-life of IgG3. Additionally, BA.1 S-specific IgG2, IgG4, IgA1, and IgM levels showed little or subtle changes over time (Supplementary Fig. 8).

FcγR-binding and Fc-mediated effector functions generally reflected the dynamics observed in antibody titers (Fig. 5d–k). Ad26.S vaccination led to robust FcγR-binding and functional responses in the Placebo-Ad26.S, BBIBP-Ad26.S, and Ad26.S-BBIBP groups (red, turquoise, and purple). Particularly, when Ad26.S priming was followed by a BBIBP boost, FcγR2A, FcγR2B, and FcγR3B-binding antibodies, as well as ADNP, ADCP, and ADNKA activities, remained significantly augmented through D196 relative to those at D0 (*P* = 0.00007, *P* = 0.005, *P* = 0.012, *P* = 0.031, *P* = 0.019, and *P* = 0.002, respectively) (Fig. 5d, e, g, i–k; purple), indicating extended long-term functional humoral immunity to the antigenically distant Omicron variant. Interestingly, FcγR2A-binding antibodies remained significantly elevated through D364 compared to those at D0 in the Ad26.S-BBIBP group (*P* = 0.0004), whereas FcγR3A-binding declined markedly from their D56 peak by D196 (Fig. 5d, f). In contrast, FcγR2A-binding antibodies persisted through D196 in the BBIBP-Ad26.S group (*P* = 0.002) and through D364 (*P* = 0.001) with sustained ADNP activity maintained until D196 (*P* = 0.004) in the Placebo-Ad26.S group. Consistent with the findings for WT-specific responses, group-wise comparisons at each time point showed stronger responses in Omicron BA.1-specific antibody levels, Fc receptor-binding and Fc-mediated effector functions in the single-dose Ad26.S and the heterologous regimens than the BBIBP-BBIBP homologous regimen following the booster dose, although no significant differences were observed during the long-term period following D56 (Supplementary Fig. 9). Taken together, these findings demonstrate that the Ad26.S-BBIBP heterologous regimen elicits robust, broad, and long-lasting antibody Fc-mediated effector functions against both the SARS-CoV-2 WT strain and antigenically drifted Omicron BA.1 variant, particularly with ADCP, ADNP, and ADNKA. The effector functions largely tracked with IgG and FcγR interactions with the S.

### BBIBP boosting attenuates the waning of immunity primed by Ad26.S

**Fig. 6:**
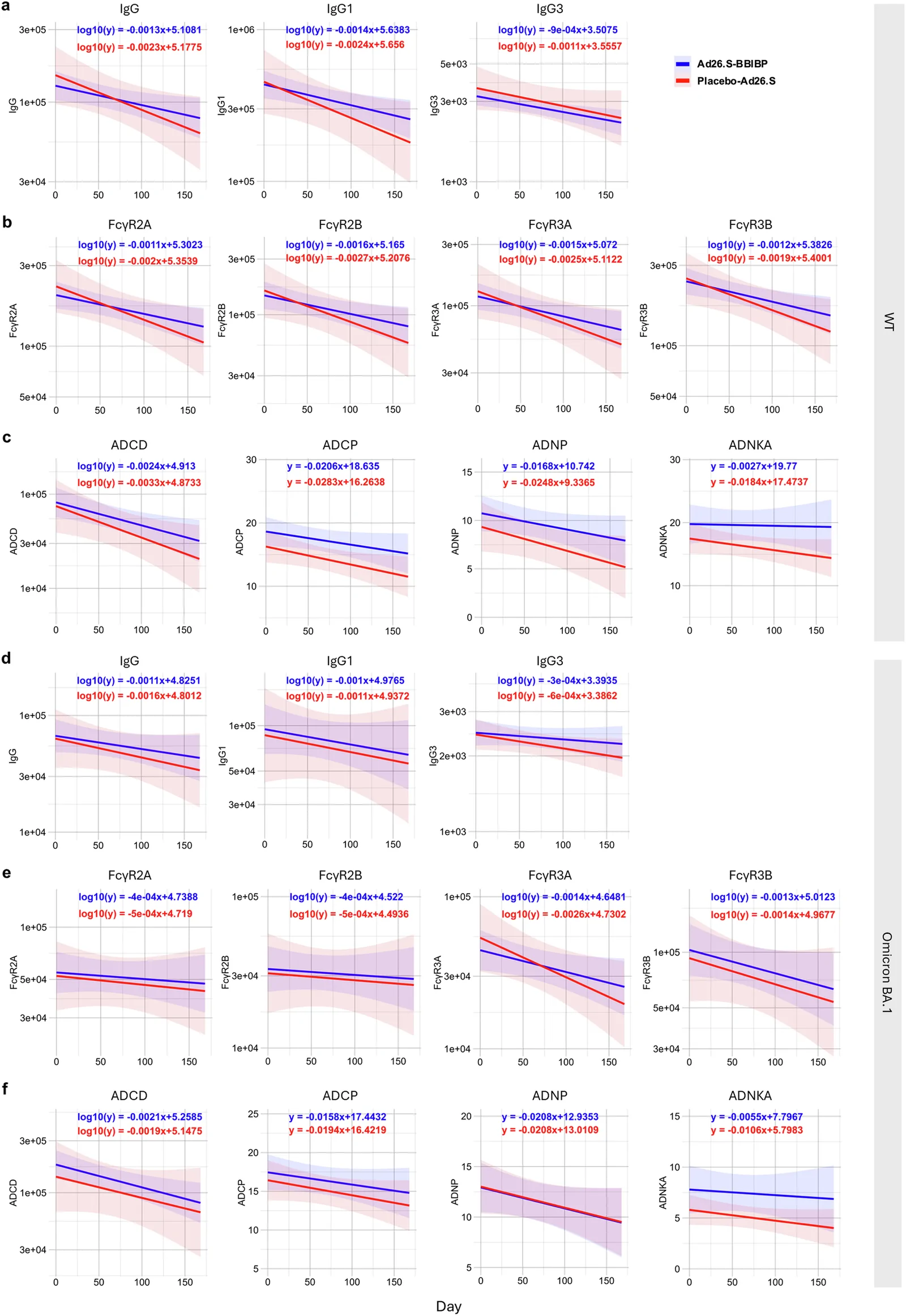
Ad26.S-BBIBP prime-boost regimen demonstrates reduced immune decay compared to placebo-Ad26.S. Waning rates of **a**, **d** antibody levels (IgG, IgG1, and IgG3), **b**, **e** FcγR binding (FcγR2A, FcγR2B, FcγR3A, and FcγR3B), and **c**, **f** functional activities (ADCD, ADCP, ADNP, and ADNKA) against WT (**a**–**c**) and BA.1 (**d**–**f**) S proteins were evaluated over a 168-day period from peak response. Placebo-Ad26.S (red) and Ad26.S-BBIBP (purple) groups were compared using log-transformed GMFI values (except for ADCP, ADNP, and ADNKA, which were expressed as percentages) via linear regression modeling. Best-fit lines with 95% confidence intervals (shaded) are shown, and the equations are color-coded for each group.

Next, we quantified immune durability and evaluated the effect of BBIBP boosting on Ad26.S priming by comparing the decay rates of immune features using linear regression modeling between the Ad26.S-BBIBP group (from peak at days 28–196) and the Placebo-Ad26.S group (from peak at days 56 to 224 to match the equivalent 168-day follow-up period) (Fig. 6). Although group-based comparisons of the half-lives of each immune feature did not reach statistical significance (data not shown), the overall decay trends appeared to differ between the two groups. Across all SARS-CoV-2 WT S-specific features, the Ad26.S-BBIBP group exhibited a more gradual decline in immune responses than the Placebo-Ad26.S group (Fig. 6a–c), indicating that BBIBP boosting helped sustain the full spectrum of WT-specific humoral responses induced by Ad26.S priming. These effects collectively contributed to the enhanced durability of vaccine-induced immunity in this regimen. For BA.1 S-specific features, decay rates were generally comparable between groups but were slower in the Ad26.S-BBIBP group for IgG3, FcγR3A-binding, and ADNKA activity (Fig. 6d–f), indicating their conserved roles in cross-variant durability. Additionally, features previously identified as being less involved in long-term immunity, such as IgG2, IgG4, IgA1, and IgM, were analyzed (Supplementary Fig. 10). However, their initially low titers and subtle temporal changes resulted in limited meaningful comparisons of decay rates. Together, these results demonstrate that BBIBP boosting helps maintain Ad26.S-primed immunity stable through D196. Notably, ADNKA was the most durably preserved Fc-mediated response, possibly elicited via IgG3 and FcγR3A interaction, across both WT and Omicron BA.1 antigens.

### ADNKA is defined as a pivotal feature of durable immunity to WT and Omicron BA.1 in Ad26.S-BBIBP recipients

**Fig. 7:**
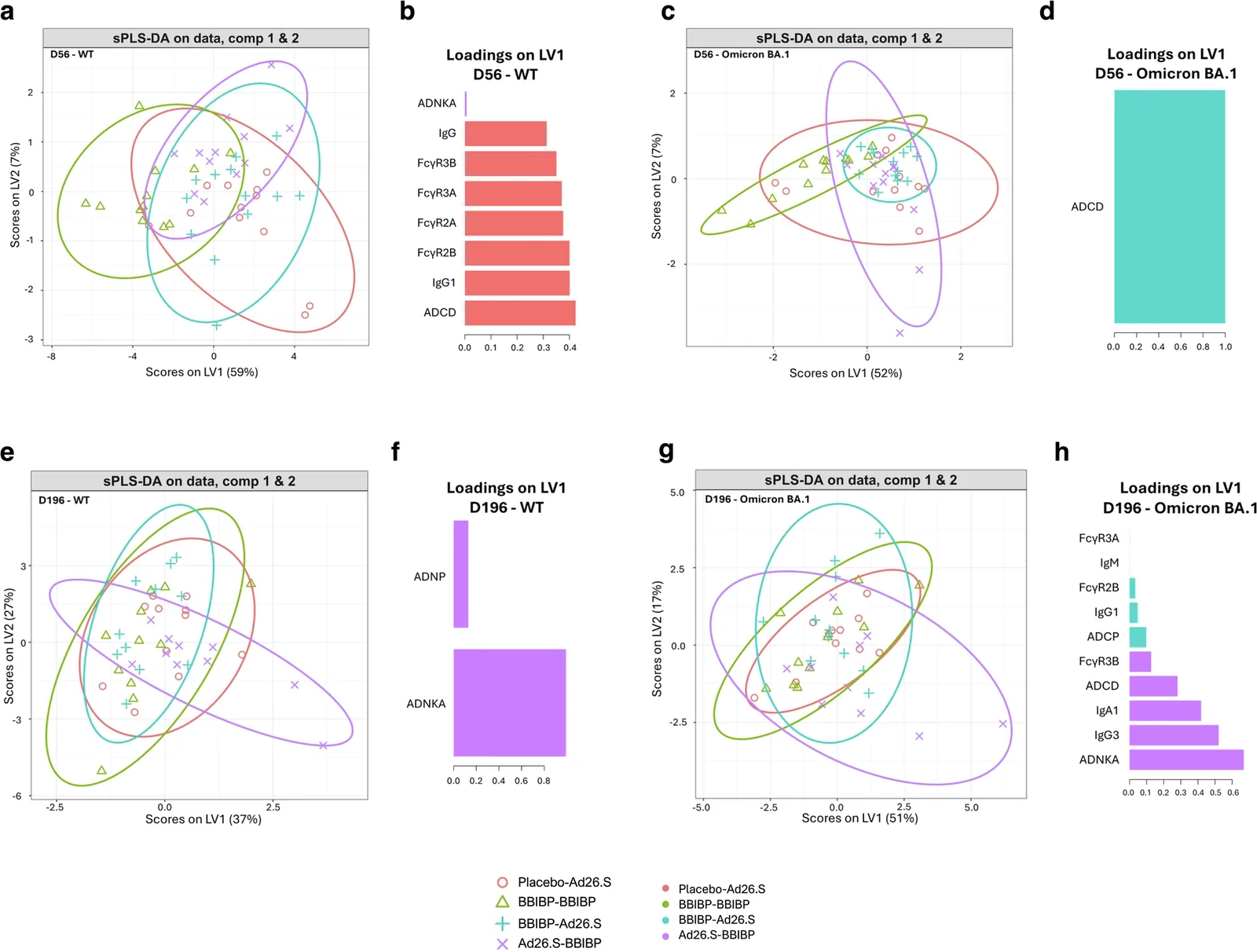
Multivariate analysis of humoral immunity at D56 and D196 across four vaccine regimens. Sparse partial least squares discriminant analysis (sPLS-DA) clustering of humoral profiles of four vaccination groups against SARS-CoV-2 WT S at **a** D56 and **e** D196, and Omicron BA.1 S at **c** D56 and **g** D196. Each point represents an individual participant. The ranked contributions of immune features to the model along latent variable 1 (LV1) are shown for WT S at **b** D56 and **f** D196, and BA.1 S at **d** D56 and **h** D196. Symbols and colors denote vaccination groups: Placebo-Ad26.S (red circles), BBIBP-BBIBP (green triangles), BBIBP-Ad26.S (turquoise pluses), and Ad26.S-BBIBP (purple crosses).

Finally, to define the most discriminative immune features underlying the observed differences among the four vaccination regimens, we performed sparse partial least squares discriminant analysis (sPLS-DA) on SARS-CoV-2 WT and BA.1 S-specific humoral immune responses at D56 and D196 (Fig. 7). Receiver operating characteristic (ROC) curves and area under the curve (AUC) values were used to assess the discriminative performance of the sPLS-DA models (Supplementary Fig. 11). At D56, the BBIBP-BBIBP regimen showed the highest discriminative performance for both WT (AUC = 0.9437) and Omicron BA.1 (0.9324) (Supplementary Fig. 11a, b). For WT, this was followed by Ad26.S-BBIBP (0.8536), Placebo-Ad26.S (0.8243), and BBIBP-Ad26.S (0.7393), whereas for Omicron BA.1, BBIBP-Ad26.S (0.765) showed higher performance than Ad26.S-BBIBP (0.6599) and Placebo-Ad26.S (0.6329). At D196, the Ad26.S-BBIBP regimen showed the highest discriminatory power (AUC = 0.8182 for WT and 0.8061 for Omicron) for both WT and Omicron BA.1 responses, followed by BBIBP-Ad26.S (0.733, 0.6705), BBIBP-BBIBP (0.7159, 0.6534), and Placebo-Ad26.S (0.5682, 0.5028) (Supplementary Fig. 11c, d).

In the sPLS-DA models, the degree of separation was less pronounced at D56 for both WT and Omicron BA.1 (Fig. 7a, c), suggesting that early differences in immune responses were not sufficiently distinct to fully discriminate between vaccine regimens. In contrast, clearer separation was observed at D196, with the Ad26.S-BBIBP group forming a distinct cluster along latent variable 1 (LV1) and LV2 for both the WT and BA.1 antigens (Fig. 7e, g). The features in the loading plots were ranked according to their importance in discriminating between groups (Fig. 7b, d, f, h; Supplementary Fig. 12). At D56, ADNKA in the Ad26.S-BBIBP group emerged as a contributory feature, although with relatively lower loading weights (Fig. 7b; Supplementary Fig. 12b), whereas most features appeared more influential in the Placebo-Ad26.S group at this early timepoint. At D196, loading patterns became more strongly dominated by features associated with the Ad26.S-BBIBP group (Fig. 7f, h). Particularly, ADNKA and ADNP were prominently elevated for WT S-specific responses (Fig. 7f) and ADNKA, IgG3, IgA1, and ADCD for Omicron BA.1 (Fig. 7h). Consistent with the univariate analyses, all these features, except for IgA1, remained significantly augmented from baseline to D196 (Figs. 2, 4, and 5), supporting their contribution to the durability of the Ad26.S-BBIBP-induced immune response. It is noteworthy that ADNKA emerged as the most prominent feature shared across both antigens among all. Together, these findings indicate that differences in humoral immune responses become more clearly defined over time. Additionally, these data highlight the central role of ADNKA in mediating cross-variant and long-term humoral immunity in the Ad26.S-BBIBP recipients and its contribution to durable protection beyond neutralization in this cohort.

## Discussion

This study provides a comprehensive analysis of the immunogenicity, durability, and functional breadth of homologous and heterologous prime-boost regimens using Ad26.S and BBIBP vaccines in cohorts from Mozambique and Madagascar. Our findings reveal how distinct prime-boost strategies differentially induce humoral responses, particularly antibody-mediated effector functions, which are crucial for protecting against severe COVID-19 and mortality^37^. Our data demonstrate that the heterologous Ad26.S-BBIBP regimen elicited a rapid, robust, and durable immune response, which was sustained for at least 6 months post-priming. This response exhibited broad activity against both the ancestral SARS-CoV-2 strain and immune-evasive Omicron BA.1 variant. Notably, among the various Fc-mediated effector functions analyzed, ADNKA was significantly enhanced and remained durable over time in the Ad26.S-BBIBP regimen group, suggesting that it contributes to the broad and long-term immune response offered by this regimen.

Although not all antibody binding directly translates into effector function^38^, the observed maintenance of the IgG1 and IgG3 subclasses and prolonged FcγR-binding in the Ad26.S-BBIBP group against both the WT strain and BA.1 variant may reflect qualitative enhancement of humoral immunity (Figs. 2, 3, and 5). These specific IgG subclasses are particularly efficient at engaging FcγR2A on neutrophils and monocytes, as well as FcγR3A on NK cells^39,40^. These results align directly with the persistent ADNP, ADCP, and ADNKA levels observed in this study. Crosslinking of FcγRs on phagocytes such as neutrophils and monocytes induces phagocytosis of IgG-opsonized virions and infected cells^39^. S-specific antibodies engaging with FcγR3A is a well-established mechanism for triggering NK cell-mediated cellular cytotoxicity for direct killing of virally infected cells^40^. These responses were observed to be more robust against the WT strain than the BA.1 variant (Figs. 5, 6), likely because both vaccines encode the original WT S protein^17,41^. Therefore, more effective immune recognition and boosting against homologous antigens might have been promoted. IgG2 and IgG4 have low binding affinity, whereas IgA and IgM have no binding affinity to all four FcγRs, supporting little to no engagement of these isotypes and subclasses in inducing effector functions^42^. In ADCD, IgG and IgM are actively engaged in complement activation by binding to C1q, leading to activation of the complement cascade^43^. Because of the low detection of IgM in our study, the persistence of ADCD in the Ad26.S-BBIBP group may be attributed to sustained levels of IgG. These collectively suggest that Ad26.S-BBIBP-induced antibodies are functionally qualitative with enhanced Fc-mediated functional activities in phagocytosis, cytotoxicity, and complement.

Throughout the study, the inactivated viral vaccine BBIBP elicited suboptimal immune responses across all measured features. This appeared consistent with prior reports indicating minimal antibody titers following a single dose and the necessity of a third dose to achieve dramatically increased IgG binding and neutralization^44,45^. Moreover, the BBIBP-Ad26.S group exhibited immune response trajectories that closely resembled those of the single-dose Ad26.S group, underscoring the limited priming capacity of BBIBP. However, BBIBP showed a promising effect when used as a booster on Ad26.S-primed immunity. These point to the critical role of Ad26.S as an effective priming agent in heterologous regimens that enhance and sustain immune responses. The benefits of utilizing replication-deficient adenoviral vectors for priming are well-documented^46,47^. Adenoviral vector vaccines, including Ad26.S, are known to induce strong Th1-skewed CD4^+^ T cell responses^17,48^ and durable humoral immunity via the generation of spike-specific memory B cells and long-lived plasma cells in lymphoid tissues^49–51^. Given the established association between these immunological features and long-term protection^52^, our data support that Ad26.S priming provides a solid immunological foundation that potentiates the effects of subsequent BBIBP boosting. These highlight the importance of priming platform selection for optimizing heterologous vaccine regimens.

Among the measured features, both the decay kinetics and sPLS-DA results indicated that ADNKA is a dominant and uniquely sustained cross-variant antibody function following Ad26.S-BBIBP vaccination, across both SARS-CoV-2 WT and the BA.1 variant. Notably, these increases and persistence appear to reflect a distinct immunological advantage that is specific to certain vaccine regimens. ChAdOx1-S/BNT162b2 combinations have been reported to reduce NK cell activation markers and potentially impair Fc-mediated effector functions^53^. In contrast, inactivated vaccines have been shown to enhance IFN-γ and CD107a expression on NK cells two weeks after vaccination and to promote the development of trained-like NK cell immunity in both humans and non-human primates^54,55^. These suggest that inactivated vaccines may contribute to the long-term maintenance of ADNKA in the Ad26.S-BBIBP regimen in our study. Given that robust NK cell-mediated effector responses are linked to favorable COVID-19 outcomes and vaccine-induced protection in non-human primates^56^, this heterologous regimen is expected to provide durable protection for SARS-COV-2 infection.

Emerging SARS-CoV-2 variants, particularly the Omicron lineage, reduces neutralization capacity following homologous Ad26.S or BBIBP vaccination due to numerous mutations accumulated in the S protein^57,58^. Heterologous boosting, however, can overcome the limitations of primary regimens by improving both the magnitude and breadth of antibody responses^59^. For instance, an mRNA vaccine booster following either an adenoviral vector-based vaccine (ChAdOx1 nCoV-19) or an inactivated vaccine (BBIBP-CorV or CoronaVac) markedly increases both humoral and cellular immune responses to SARS-CoV-2 VOCs^14,15^. While both ChAdOx1 and BNT162b2 boosters enhanced immunity in ChAdOx1-primed individuals, BNT162b2 induced stronger spike-specific T-cell responses and higher NAb titers against multiple variants. Similarly, two doses of an inactivated vaccine followed by an mRNA booster elicited robust RBD- and S-specific antibody levels and neutralization titers that were significantly higher than those from three homologous inactivated doses, including against Omicron variant. In our study, the heterologous Ad26.S-BBIBP regimen elicited stronger and more durable NAb responses, together with enhanced Fc-mediated functions, compared with those of single-Ad26.S and homologous BBIBP regimens. sPLS-DA results specifically revealed that Fc-mediated functions are effective in discriminating groups (Fig. 7), indicating that these functions support explaining the distinctive dynamics observed in each vaccine regimen in addition to NAb titers. Despite the expected response reduction in BA.1, Fc-mediated effector functions, specifically ADNKA and its related features, remained elevated against both antigens following Ad26.S-BBIBP vaccination. Collectively, these underscore the advantage of Ad26.S-BBIBP heterologous vaccination in broadening durable immune protection across SARS-CoV-2 variants through both neutralizing and non-neutralizing pathways.

Indeed, in a previous interim analysis conducted in a larger main cohort (*n* = 367), the assessment of Omicron BA.1-specific neutralizing activity showed NAb titers trajectories that were highly similar to our systems serology data^34^. For instance, following BBIBP vaccination, NAb titers increased only marginally, whereas at the time point after Ad26.S vaccination, the geometric mean titers (GMT) of NAbs increased by at least threefold across all vaccination groups. Meanwhile, the absolute levels of the GMT were affected depending on the serostatus, yet the overall patterns of GMT changes over time were comparable between the groups. These support that our antibody and functional outcomes, as well as immune response duration, are less likely to be affected by serostatus.

This study had some limitations. First, the inclusion of both seropositive and seronegative individuals at baseline complicated attribution of immune responses solely to vaccination. To overcome this limitation, we attempted to evaluate whether pre-exposure skewed the serological analyses. However, the number of seronegative individuals within each vaccination group at each time point was very limited, ranging from one to four participants per group, which precluded statistically meaningful analyses. Additionally, although samples with markedly elevated nucleoprotein-specific IgG (NP-IgG) levels were excluded, the possibility of undetected breakthrough infections remained. Second, the single Ad26.S group had a limited role as a control, since the dose was administered only at the booster time point in the prime-boost sequence. A more informative longitudinal comparison with Ad26.S-BBIBP could have been achieved if Ad26.S-Placebo arm had been included. We addressed this limitation by matching the time periods between peak and follow-up periods in decay modeling. This step revealed more gradual-waning kinetics of the Ad26.S-BBIBP regimen relative to the single Ad26.S group. Nevertheless, optimized study designs are required to enable direct, time-matched comparisons across groups.

Further research involving extensive cellular functional assays and detailed mechanistic analyses will be insightful. Given the robust and durable T-cell response induced by adenoviral vector vaccines^60,61^, exploring the cellular facets of vaccine-induced immunity will help fully understand the effect of this heterologous regimen on long-term protection. Concurrently, glycan profiling and multi-omics analysis will provide precise validation of specific Fc-mediated pathways as well as a holistic understanding of host–pathogen interactions and immune regulation^62^. Moreover, investigating the dynamics of memory-like NK cells may reveal their contribution to long-term protection and the unique features of these heterologous combinations. Lastly, assessing vaccine regimens against the latest circulating Omicron variants may support the up-to-date practicality of this vaccination strategy. These integrated approaches may provide crucial insights not only for rational vaccine design but also for optimizing future vaccination strategies, particularly in the context of emerging variants.

In conclusion, using systems serology, we comprehensively characterized vaccine-induced humoral immune responses and highlighted the importance of nNAb functions along with neutralization in conferring protection. Our findings demonstrate that the Ad26.S-BBIBP heterologous regimen elicits robust, broad, and durable antibody Fc-mediated effector functions against both the SARS-CoV-2 WT strain and Omicron BA.1 variant, with sustained ADNKA as a key cross-variant feature unique to this regimen. By leveraging the logistical advantages of the two vaccines, this heterologous regimen represents a potentially cost-effective, practical and scalable vaccination strategy for long-term protection with broader variant coverage, particularly in regions where frequent boosting is not feasible.

## Methods

### Study design

This study was conducted as part of a Phase II, multicenter, observer-blinded, randomized, non-inferiority clinical trial (ClinicalTrials.gov Identifier: NCT04998240)^33^ to explore the Fc-mediated effector responses induced by homologous and heterologous COVID-19 vaccine regimens. A total of 367 healthy adults aged 18–65 years were enrolled from clinical sites in Mozambique and Madagascar (Table 1). Participants were assigned to one of four arms: 1) Placebo-Ad26.S, 2) BBIBP-BBIBP, 3) BBIBP-Ad26.S, or 4) Ad26.S-BBIBP. The study population consisted of a main cohort (*n* = 300; 75 participants per group) designated for assessing primary immunogenicity endpoints, along with an immunology subset comprising 60 participants drawn from the main cohort, with 14–16 individuals per group, intended for extended exploratory analyses. Serum samples for the present analysis were obtained exclusively from the immunology subset. Participants received a prime dose on D0 and a booster dose on D28 via intramuscular injection, with follow-up for 12 months. Blood samples were collected on days 0, 28, 56, 196, and 364.

The eligible participants included individuals with well-controlled mild-to-moderate comorbidities. Key exclusion criteria were prior COVID-19 vaccination, active SARS-CoV-2 infection at enrollment, known infection with hepatitis B and C, notable clinical abnormalities, bleeding disorders, ongoing anticoagulant therapy, recent receipt of immunoglobulins or blood products, known allergies to vaccine components, and current or planned pregnancy or lactation. Following the completion of multiplex and functional assays, NP-IgG levels in the main cohort were measured using an enzyme-linked immunosorbent assay. To minimize potential breakthrough infections and better isolate vaccine-induced immune responses, samples with markedly elevated NP-IgG levels, indicative of potential breakthrough infections^63^, were excluded from analysis (Supplementary Tables 3 and 4). To establish the exclusion threshold, the fold-changes in NP-IgG levels between time points were calculated for each sample in the main cohort. To specifically capture significant increases, fold-change values of less than 1 were excluded. The 1.5× interquartile range rule was applied to identify outliers^64^, with the upper threshold defined as Q3 + 1.5× interquartile range, which corresponds to a fold-change of 11.86. All samples exceeding this value and subsequent samples from the same participant were excluded from the final dataset.

### Serum sample preparation

All frozen serum samples were thawed at 20–25 °C and heat-inactivated at 56 °C for 30 min using heat blocks. Samples were diluted in phosphate-buffered saline (PBS) and stored at −80 °C.

### Antigen-coupling with beads

Antigen-bead coupling was performed following a previously established protocol for assays utilizing the WT SARS-CoV-2^65^. For both the functional and Luminex assays targeting the Omicron VOC, the B.1.1.529 (Omicron) S1 + S2 trimer protein (#40589-V08H26, Sino Biological) served as an antigen. A single type of Luminex Magplex-Avidin microsphere (#MA-A012-01) was used for Luminex assays.

### ADCD

The ADCD assays were conducted as previously described^65^. Red fluorescent NeutraAvidin-coated beads were conjugated to biotinylated SARS-CoV-2 antigens. Beads coupled with either the original strain or B.1.1.529 variant antigens were incubated with plasma samples diluted 1:40 and 1:20 in PBS, respectively, at 37 °C for 2 h to allow immune complex formation, followed by washing. The lyophilized guinea pig complement (#CL4051, Cedarlane, Burlington, Ontario, Canada) was reconstituted in RPMI 1640 medium (#11875119, Gibco, Grand Island, NY, USA) supplemented with 10% fetal bovine serum (FBS; #16000044, Gibco) and then added to the washed immune complexes. The mixture was incubated for 50 min at 37 °C. Complement component C3 deposition was detected by staining with anti-C3 fluorescein isothiocyanate-conjugated goat IgG (#0855385, MP Biomedicals, Santa Ana, CA, USA). After staining for 15 min, the samples were fixed with 0.5% paraformaldehyde (#554655, BD Biosciences, Franklin Lakes, NJ, USA) in PBS and analyzed using iQue3 flow cytometry (Sartorius, Göttingen, Germany) to assess the geometric mean fluorescence intensity (GMFI) of fluorescein isothiocyanate-labeled C3 deposition.

### ADCP

The ADCP was conducted as previously described^65^ with minor modifications to the cell incubation time. Yellow–green fluorescent NeutraAvidin-coated beads were conjugated to biotinylated SARS-CoV-2 antigens. These antigen-bead complexes were incubated with serum, diluted at 1:800 for the original SARS-CoV-2 strain and 1:320 for the B.1.1.529 variant, in PBS at 37 °C for 2 h, followed by washing with PBS. The immune complexes formed were added to wells containing 2.5 × 10^4^ THP-1 cells (#TIB-202, ATCC, Manassas, VA, USA) in THP-1 medium (RPMI 1640 medium supplemented with 10% FBS and 1.14% antibiotic–antimycotic). The mixture was incubated for 4 h at 37°C with 5% CO_2_. The cells were then fixed using 0.5% paraformaldehyde in PBS and analyzed using iQue3. Phagocytic scores were determined by multiplying the percentage of THP-1 cells that had internalized fluorescent beads by the mean fluorescence intensity (MFI) of beads phagocytosed by each cell and normalized per million (% bead-containing cells × MFI bead-positive/1,000,000).

### ADNP

The ADNP assays were performed as previously described^65^. Yellow–green fluorescent NeutraAvidin-coated beads were conjugated to biotinylated SARS-CoV-2 antigens. These antigen-bead constructs were incubated with serum samples, diluted 1:80 for the original SARS-CoV-2 strain and 1:40 for the Omicron B.1.1.529 variant, in PBS at 37 °C for 2 h, and then washed with PBS to remove unbound components. The resulting immune complexes were incubated with 5.0 × 10⁴ HL-60 cells (#CCL-240, ATCC) per well in HL-60 medium, consisting of RPMI 1640 supplemented with 11.4% FBS, 1.14% GlutaMax I (#35050–061, Gibco), and 1.14% antibiotic–antimycotic. The cells were then incubated at 37°C with 5% CO₂ for 1 h. Prior to this, HL-60 cells were differentiated for 6 days in HL-60 differentiation medium composed of RPMI 1640 with 0.91% dimethyl sulfoxide (#D2650, Sigma-Aldrich, St. Louis, MO, USA), 11.4% FBS, and 1.14% GlutaMax I. Following incubation with immune complexes, the cells were stained with CD11b antibody (#555388, BD Biosciences) at a 1:3 dilution in PBS and incubated for 15 min. The cells were then washed with PBS and fixed with 0.5% paraformaldehyde in PBS. Antibody-mediated phagocytosis was quantified using iQue3 flow cytometer. Phagocytic scores were determined by multiplying the percentage of neutrophils containing fluorescent beads by the MFI of beads in each cell and normalized per million cells (% bead-containing cells × MFI bead-positive/1,000,000).

### ADNKA

The ADNKA assays were conducted according to a previously described protocol^65^. A 96-well enzyme-linked immunosorbent assay plate (#439454, Thermo Fisher Scientific, Waltham, MA, USA) was coated with 30 µg of SARS-CoV-2 antigens and incubated at 37 °C for 2 h. After coating, the wells were blocked with 5% BSA in PBS and stored overnight at 4 °C to ensure proper blocking. The following day, serum samples diluted 1:80 in PBS for both the original SARS-CoV-2 strain and B.1.1.529 variant were added to the antigen-coated wells and incubated at 37 °C for 2 h, followed by thorough washing with PBS to remove unbound antibodies. NK-92 cells (#PTA-8836, ATCC) were cultured in NK cell medium (#5150, StemCell, Vancouver, Canada) supplemented with 10% horse serum (#16050-122, StemCell), 0.02 µg/mL IL-2 IS (#130-097-744, Miltenyi Biotec), and 1% penicillin–streptomycin (#15140122, Invitrogen). To detect activation markers, the medium was supplemented with an anti-CD107a-PE-Cy5 antibody (#555802, BD Biosciences), brefeldin A (5 mg/mL in dimethyl sulfoxide, #B6542, Sigma), and GolgiStop (#554724, BD Biosciences). NK-92 cells were seeded at 5 × 10⁴ cells per well and incubated for 5 h at 37 °C in a humidified incubator with 5% CO₂. After incubation, the cells were transferred to a 96-well V-bottom plate, fixed, and permeabilized using a fixation/permeabilization kit (#554714, BD Biosciences). The samples were analyzed using iQue3 flow cytometer, with gating applied to identify singlets and CD107a-positive cells. The percentage of CD107a expression was used to quantify NK cell activation.

### Antibody and FcR profiling

Antigen-specific antibodies and FcR profiling were performed as previously reported^65^. Biotinylated SARS-CoV-2 antigens, including the original SARS-CoV-2 full-length S, S1 and S2 subdomains, and RBD, as well as the Omicron BA.1 S, were attached to Luminex Magplex-Avidin microspheres. The microspheres were blocked and pooled in assay buffer prior to seeding into 96-well U-bottom plates (#34196, SPL Life Sciences, Gyeonggi-do, Korea) at a concentration of 4000 antigen-coated beads per well. Serum samples were diluted in PBS with dilution factors optimized for each antibody isotype and FcR detection assay. For the prototype SARS-CoV-2 antigens, the serum dilutions were as follows: IgG (1:640), IgG1 (1:160), IgG2 (1:160), IgG3 (1:160), IgG4 (1:80), IgM (1:40), IgA1 (1:160), FcγR2A (1:160), FcγR2B (1:80), FcγR3A (1:160), and FcγR3B (1:160). For Omicron BA.1 antigens, the dilutions were as follows: IgG (1:640), IgG1 (1:160), IgG2 (1:40), IgG3 (1:80), IgG4 (1:40), IgM (1:80), IgA1 (1:160), FcγR2A (1:160), FcγR2B (1:80), FcγR3A (1:160), and FcγR3B (1:80). The diluted serum samples were incubated with antigen-conjugated beads on a plate shaker at 650 rpm for 2 h. After incubation, the plates were washed with 0.05% Tween-20 in PBS. Antibody isotype detection was performed using isotype-specific stains (Southern Biotech, Birmingham, AL, USA and BD Pharmingen, San Diego, CA, USA), diluted in assay buffer as follows: IgG (#9040-09) at 1:200, IgG1 (#9054-09) at 1:1,000, IgG2 (#9070-09) at 1:200, IgG3 (#9210-09) at 1:200, IgG4 (#9200-09) at 1:200, IgA1 (#9130-09) at 1:200, and IgM (#562030) at 1:5. For FcR detection, streptavidin-PE (#PJ315S-1, Agilent Technologies, Santa Clara, CA, USA) was used to label Fc receptors (FcγR2A #10374-H27H1-B, FcγR2B #10259-H27H-B, FcγR3A #10389-H27H1-B, and FcγR3B #11046-H27H-B; Sino Biological, Beijing, China), diluted in assay buffer. The plates were incubated at 20 °C to 25 °C for 1 h on a shaker (650 rpm), washed again with 0.05% Tween in PBS, and analyzed using iQue3. The results are reported as the GMFI of phycoerythrin for each sample.

### Focus reduction neutralization test

Vero cells (ATCC #CCL-81) were cultured in Dulbecco’s Modified Eagle Medium (DMEM; #11995065, Invitrogen, Carlsbad, CA, USA) supplemented with 10% FBS (#26140-079, Gibco) and 1% antibiotic–antimycotic (#15240062, Gibco). Cells were seeded into 96-well flat-bottom plates (#167008, Thermo Fisher Scientific) at a concentration of 2.0 × 10⁴ cells per well and incubated overnight at 37 °C in a 5% CO₂ atmosphere. For the neutralization assay, serum samples were serially diluted threefold in DMEM containing 2% FBS. Concurrently, SARS-CoV-2 viral stocks were diluted to a final concentration of 6.0 × 10³ focus-forming units (FFU)/mL. The virus (180 FFU per well) was combined with the diluted serum in 96-well U-bottom plates (#34196, SPL Life Sciences) and incubated at 37 °C for 30 min under 5% CO₂ to allow immune complex formation. The previously plated Vero cells were rinsed with serum-free DMEM and inoculated with 150 FFU per well of the virus-serum mixture. The plates were incubated for 5 h at 37 °C in a humidified 5% CO₂ incubator. Post-infection, cells were washed with PBS and fixed overnight at 4 °C using 10% formalin (#HT501128-4L, Sigma-Aldrich). The following day, permeabilization was performed with ice-cold 100% methanol for 10 min (#32213–1 L, Sigma-Aldrich). After rinsing with PBS, the plates were blocked for 30 min at room temperature with blocking buffer (PBS, 1% BSA (#A3803-100G, Sigma-Aldrich), 0.5% goat serum (#Ab7481, Abcam, Cambridge, UK), and 0.5% Tween-20 (#T9100-100, GenDEPOT, Houston, TX, USA)). The plates were then incubated at 37°C for 1 h with a 1:3000 dilution of a rabbit monoclonal antibody specific to the SARS-CoV-2 nucleoprotein (#40143-R001, Sino Biological) in blocking buffer. Following washing with washing buffer (PBS containing 0.5% Tween-20), a secondary goat anti-rabbit IgG-horseradish peroxidase antibody was applied at a 1:2000 dilution and incubated for 1 h at 37°C. After thoroughly washing the plates, a True Blue substrate (#5510-0030, SeraCare, Milford, MA, USA) was added for signal development. A final rinse with tap water was performed, and the foci were visualized and quantified using Cytation 7 imaging system (BioTek, Winooski, VT, USA). SoftMax Pro GxP software version 7.1.2 was adopted to determine NAb titers.

### Quantification and statistical analysis

All analyses and data visualizations were conducted using R (version 4.5.0). To visualize the relative feature levels across vaccine groups, we generated a heatmap based on z-score normalization, facilitating meaningful comparisons among features and vaccine groups measured on different scales. For each feature, the log-transformed values were standardized by subtracting the mean and dividing by the standard deviation across all samples, resulting in z-scores. Violin plots were generated using ggplot showing the median for each group as solid black lines. Wilcoxon signed-rank test was performed for time point comparisons within groups (significance levels: *P < 0.05, **P < 0.01, ***P < 0.001, and ****P < 0.0001). All values were log10-transformed, except those of ADCP, ADNP, and ADNKA. We applied sPLS-DA using the ‘splsda’ function from the ‘mixOmics’ R package^66^ to identify features that most effectively distinguish among vaccine groups. To ensure the robustness of feature selection and component tuning, a ten-fold cross-validation was employed and repeated ten times.

### Ethics and regulatory statement

The trial was approved by the Institutional Review Boards of the National Institute of Health of Mozambique, International Vaccine Institute, National Ethics Committees of Mozambique and Madagascar, and National Regulatory Authority of Mozambique. The study was conducted in accordance with the Declaration of Helsinki, International Ethical Guidelines set by the Council for International Organizations of Medical Sciences, and ICH-GCP standards. All participants provided written informed consent prior to sample collection. This study was registered at ClinicalTrials.gov under the identifier NCT04998240.

## Supplementary information

Supplementary information

Supplementary Table 1

Supplementary Table 2

Supplementary Table 3

Supplementary Table 4

## Data Availability

Data supporting the findings of this study are available within the paper and supplementary materials. Any additional information can be provided by the corresponding author upon reasonable request.

## Code availability

The codes and scripts used for statistical analyses are available upon request from the corresponding author. Analyses were performed using R (version 4.5.0). All relevant packages and parameter settings are described in the Methods section.
