# Supplementary material for: Long-term cross-variant Fc-mediated immune responses against SARS-CoV-2 induced by a heterologous adenoviral/inactivated virus prime-boost vaccination strategy
Source: NPJ Vaccines. 2026 May 22;11:180. doi: 10.1038/s41541-026-01483-z (PMC13572433; doi:10.1038/s41541-026-01483-z)
Supplement: Supplementary file 1 [file 41541_2026_1483_MOESM1_ESM.pdf]

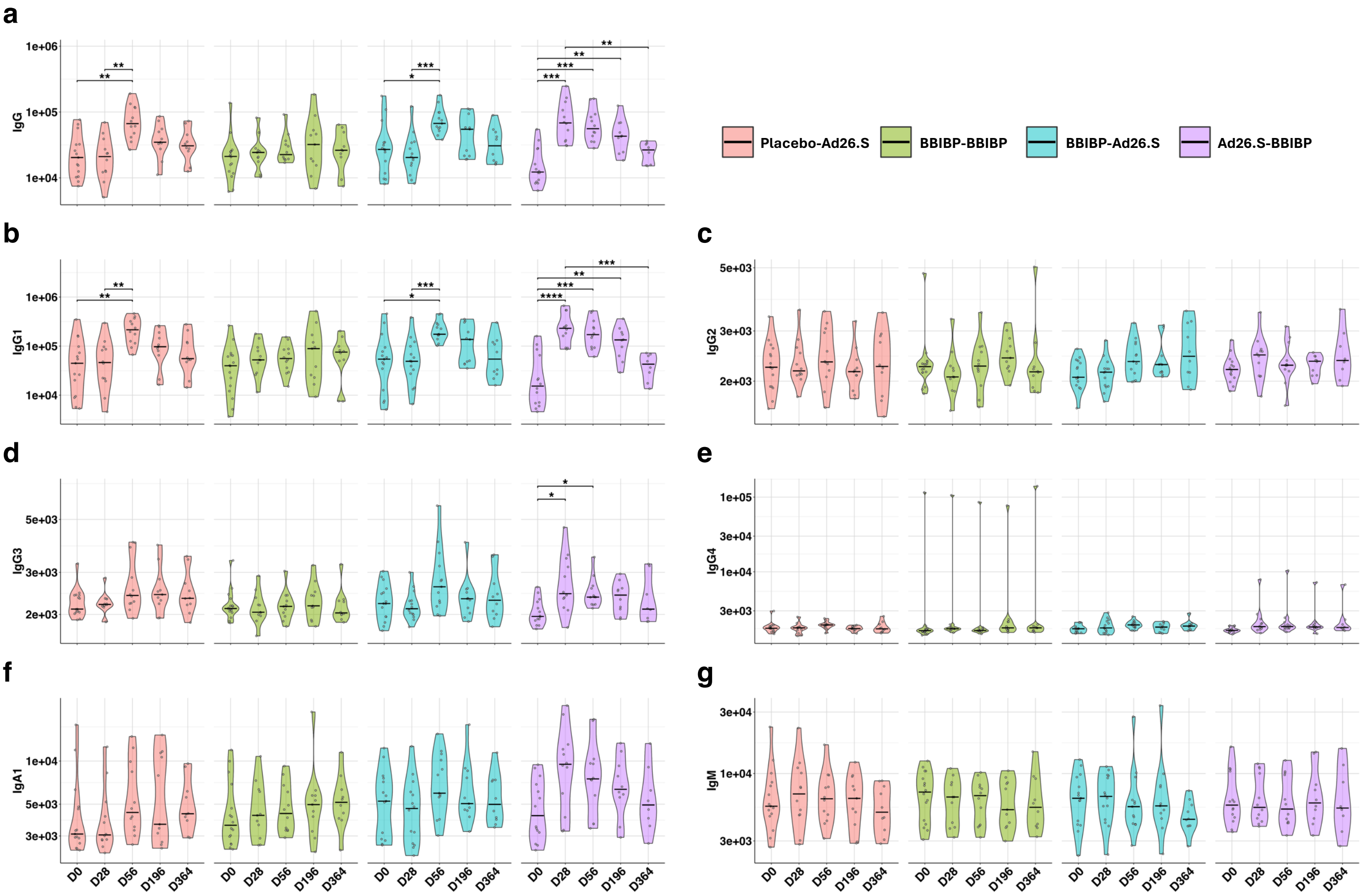

Supplementary Figure 1 SARS-CoV-2 WT S1-specific antibody isotype and subclass responses across vaccine regimens

Violin plots illustrate the longitudinal changes in SARS-CoV-2 WT S1-specific antibody responses up to D364 across the four vaccine groups. Individual measurements are shown as gray dots, with the medians represented by black lines. The y-axes display the geometric mean fluorescence intensity (GMFI) of **(a)** IgG, **(b)** IgG1, **(c)** IgG2, **(d)** IgG3, **(e)** IgG4, **(f)** IgA1, and **(g)** IgM. Each assay was performed in quadruplicate. Within each group, statistical comparisons across time points were analyzed using Wilcoxon signed-rank test with Bonferroni correction (*P < 0.05, **P < 0.01, ***P < 0.001, and ****P < 0.0001). Significance was determined only for the comparison between D0 and D28.

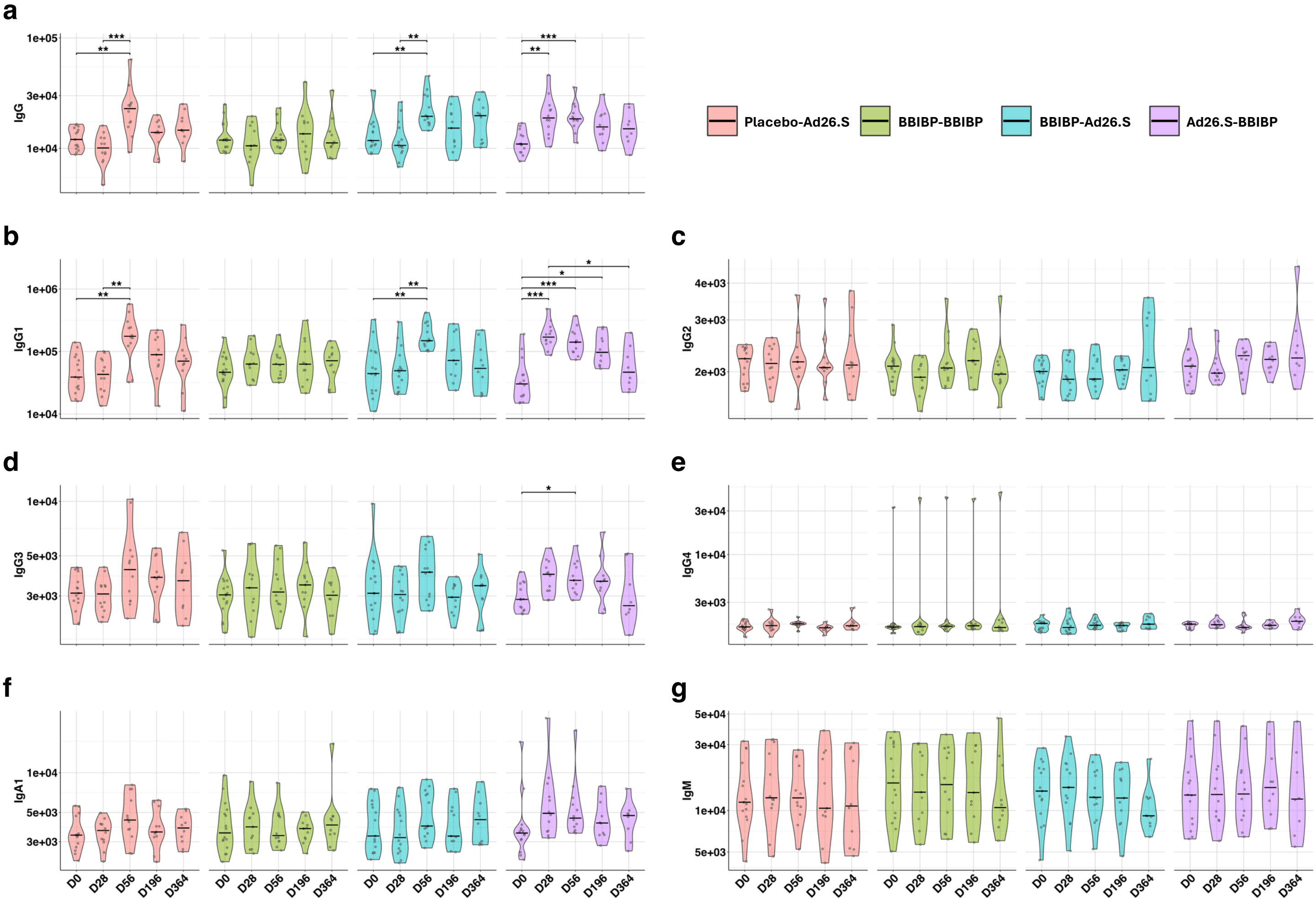

Supplementary Figure 2 SARS-CoV-2 WT S2-specific antibody isotype and subclass responses across vaccine regimens

Violin plots depict the temporal dynamics of SARS-CoV-2 WT S2-specific antibody responses measured up to D364 in the four vaccine cohorts. Individual data points appear in gray, whereas median values are marked by black dashes. The y-axes represent the GMFI of **(a)** IgG, **(b)** IgG1, **(c)** IgG2, **(d)** IgG3, **(e)** IgG4, **(f)** IgA1, and **(g)** IgM. All experiments were conducted with four technical replicates. Statistical differences between time points within each vaccine group were assessed using Wilcoxon signed-rank test and adjusted using Bonferroni correction (*P < 0.05, **P < 0.01, ***P < 0.001, and ****P < 0.0001). Statistical significance is highlighted only for comparisons between D0 and D28.

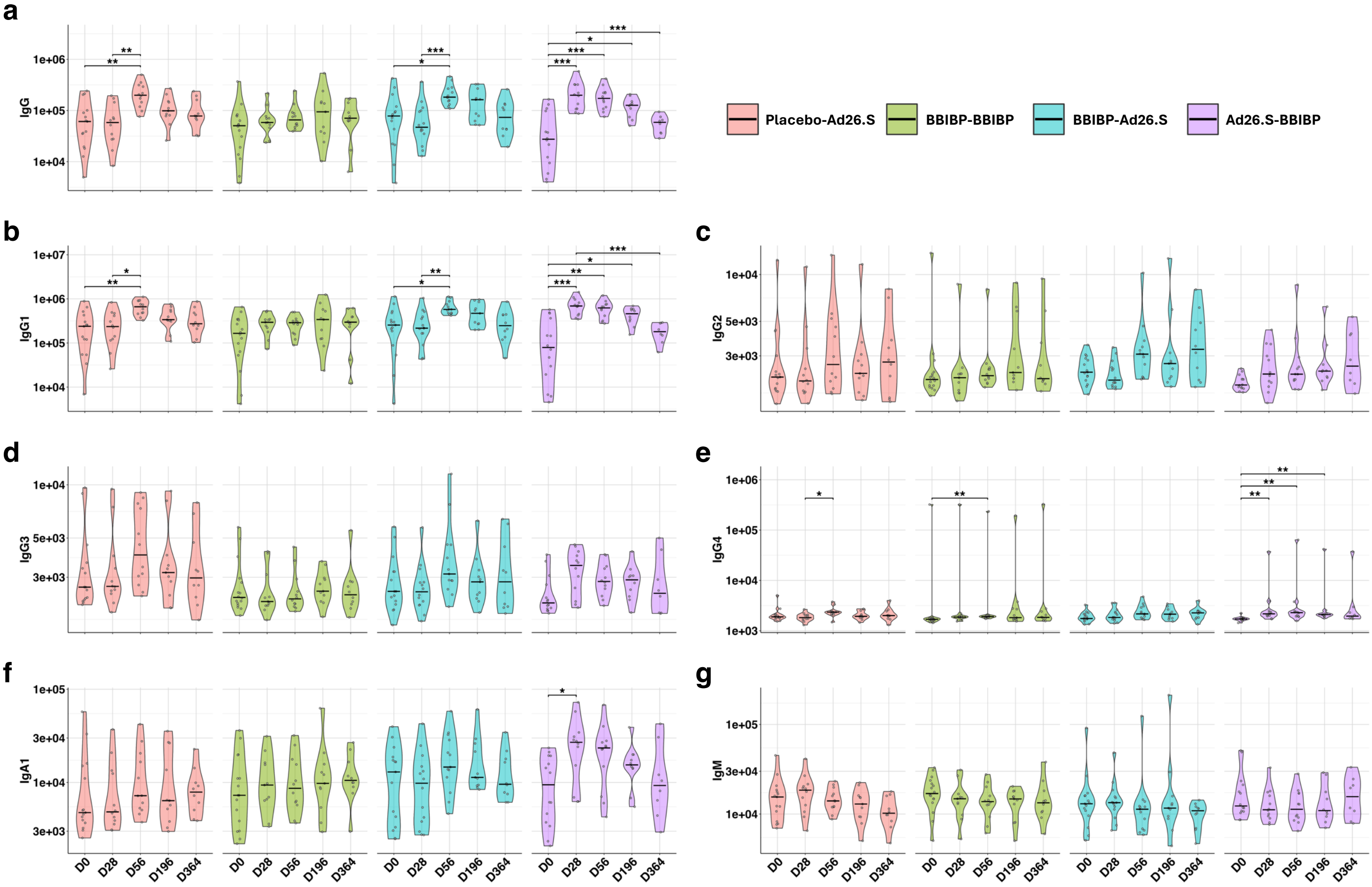

Supplementary Figure 3 SARS-CoV-2 WT RBD-specific antibody isotype and subclass responses across vaccine regimens

Longitudinal violin plots showing the SARS-CoV-2 WT RBD-specific antibody response profiles over a 364-day period for the four vaccine groups. Each gray dot represents an individual sample, and the median is indicated by a black horizontal line. The y-axes denote the GMFI of **(a)** IgG, **(b)** IgG1, **(c)** IgG2, **(d)** IgG3, **(e)** IgG4, **(f)** IgA1, and **(g)** IgM. All assays were performed in quadruplicate to ensure technical consistency. Comparisons of antibody levels between time points within each group were evaluated using Wilcoxon signed-rank test with Bonferroni adjustment (*P < 0.05, **P < 0.01, ***P < 0.001, and ****P < 0.0001). Only comparisons relative to D0 and D28 as baselines were annotated as significant.

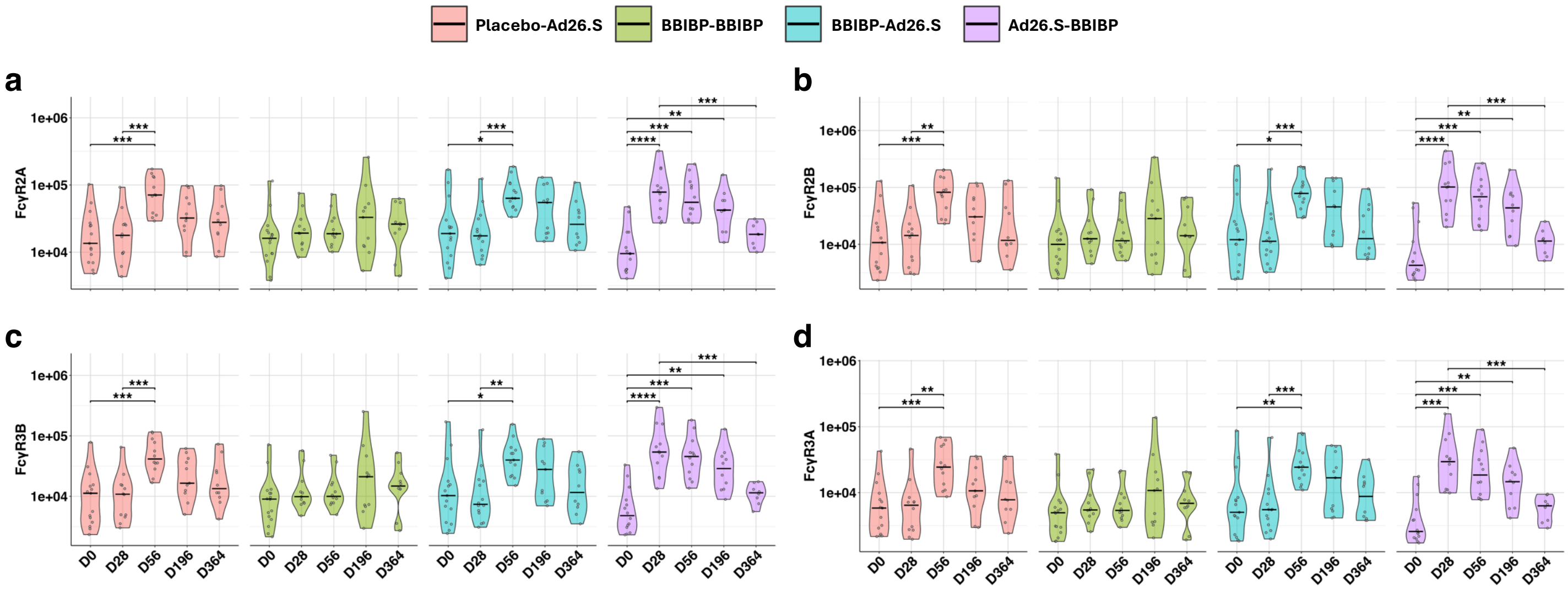

Supplementary Figure 4 SARS-CoV-2 WT S1-specific FcγR-binding antibody responses

Violin plots illustrate the longitudinal FcγR-binding antibody responses to the SARS-CoV-2 WT S1 protein measured up to D364 across four vaccine groups. Individual data points are shown as gray dots, with medians indicated by a black dashed line. The y-axes represent GMFI of **(a)** FcγR2A-, **(b)** FcγR2B-, **(c)** FcγR3A-, and **(d)** FcγR3B-binding antibodies. All assays were performed in quadruplicate. Within each group, comparisons at different time points were performed using Wilcoxon signed-rank tests with Bonferroni correction (*P < 0.05, **P < 0.01, ***P < 0.001, and ****P < 0.0001). Statistical differences are shown only in relation to D0 and D28.

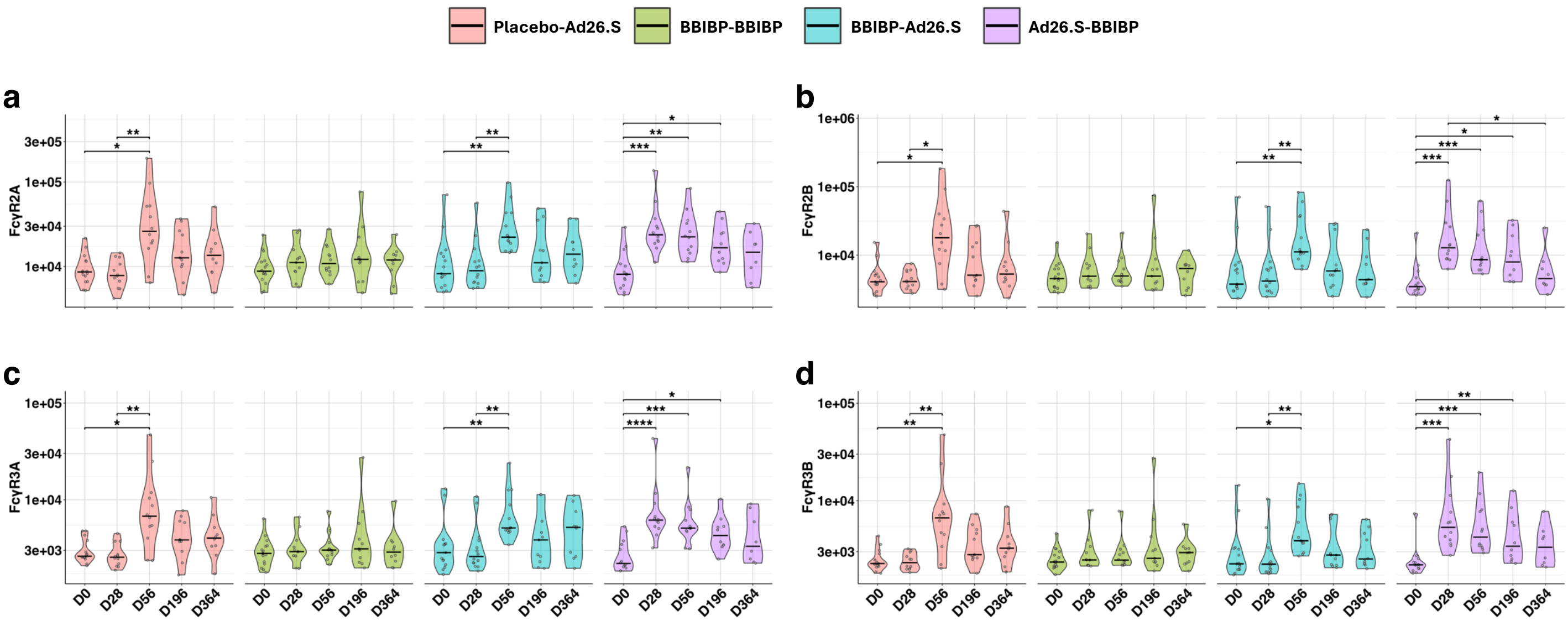

Supplementary Figure 5 SARS-CoV-2 WT S2-specific FcγR-binding antibody responses

The violin plots display longitudinal Fcγ receptor binding antibody levels against the SARS-CoV-2 WT S2 antigen up to D364 across four vaccine regimens. Gray dots represent individual measurements and black dashes denote the median values. The y-axes show GMFI values for antibodies binding to **(a)** FcγR2A, **(b)** FcγR2B, **(c)** FcγR3A, and **(d)** FcγR3B. All tests were performed in quadruplicate. Statistical comparisons between time points within each group were performed using Wilcoxon signed-rank tests with Bonferroni adjustments (*$P < 0.05$, **$P < 0.01$, ***$P < 0.001$, and ****$P < 0.0001$). Only comparisons between D0 and D28 were annotated using significance markers.

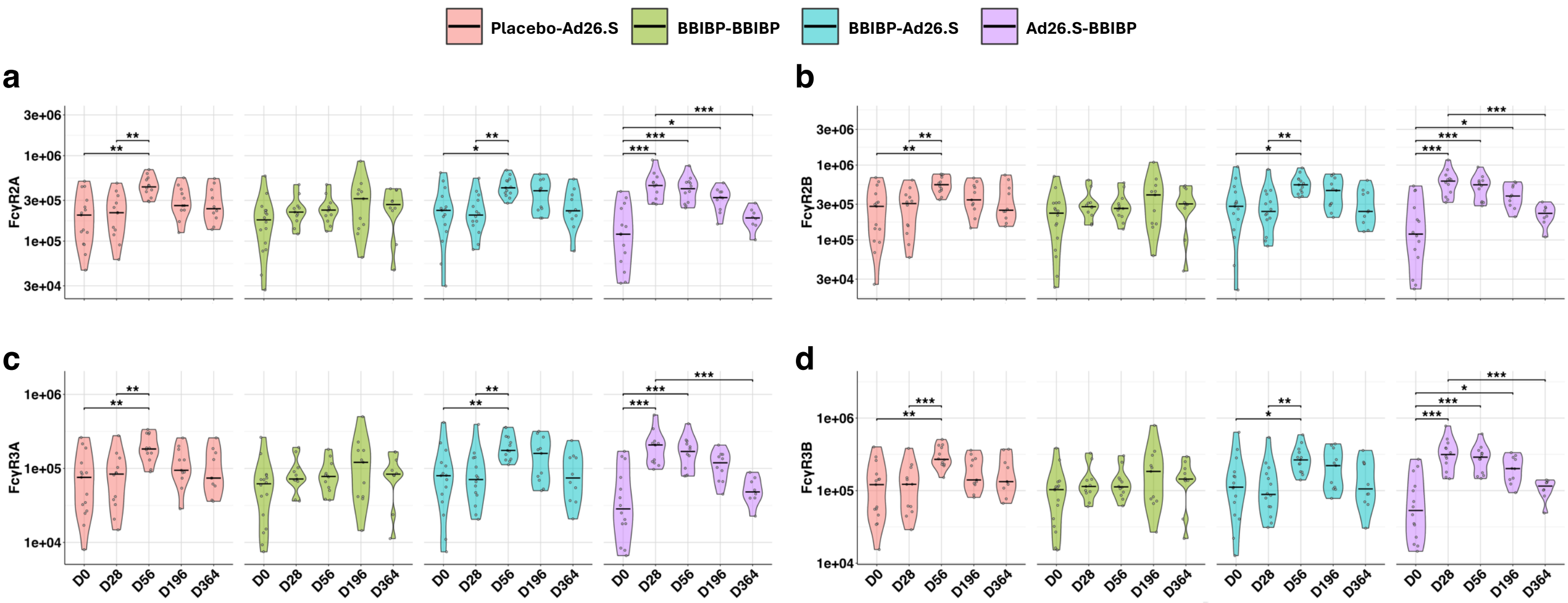

Supplementary Figure 6 SARS-CoV-2 WT RBD-specific FcγR-binding antibody responses

Longitudinal Fcγ receptor-binding antibody responses to SARS-CoV-2 WT RBD protein are shown in violin plots up to D364 for four vaccine groups. Individual results are plotted as gray dots with medians indicated by black dashed lines. The y-axes present GMFI of **(a)** FcγR2A-, **(b)** FcγR2B-, **(c)** FcγR3A-, and **(d)** FcγR3B-binding antibodies. Each assay was performed in quadruplicate. Paired comparisons over time within groups utilized Wilcoxon signed-rank tests with Bonferroni correction (*P < 0.05, **P < 0.01, ***P < 0.001, and ****P < 0.0001). Annotations of statistical significance were limited to comparisons between D0 and D28.

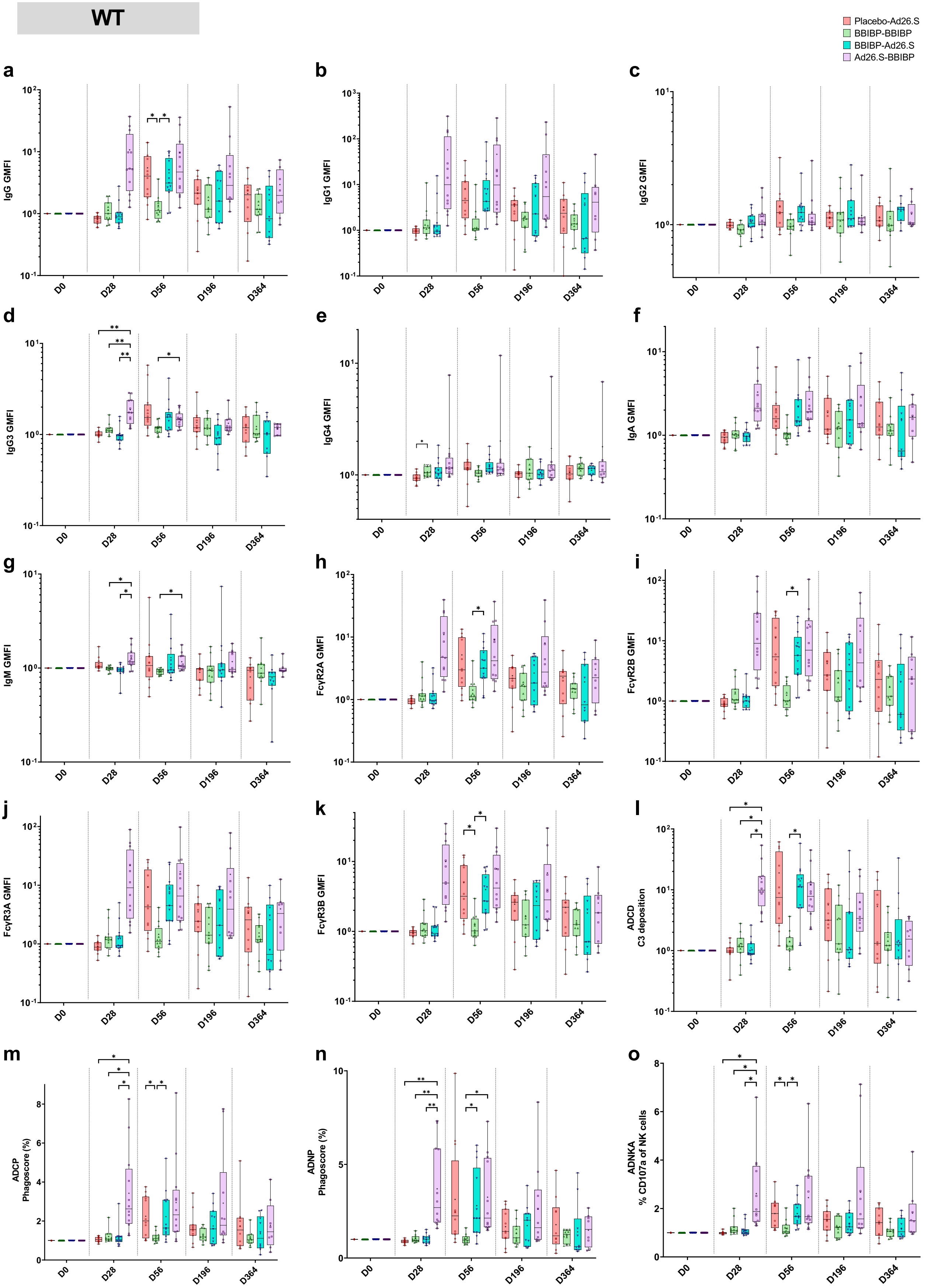

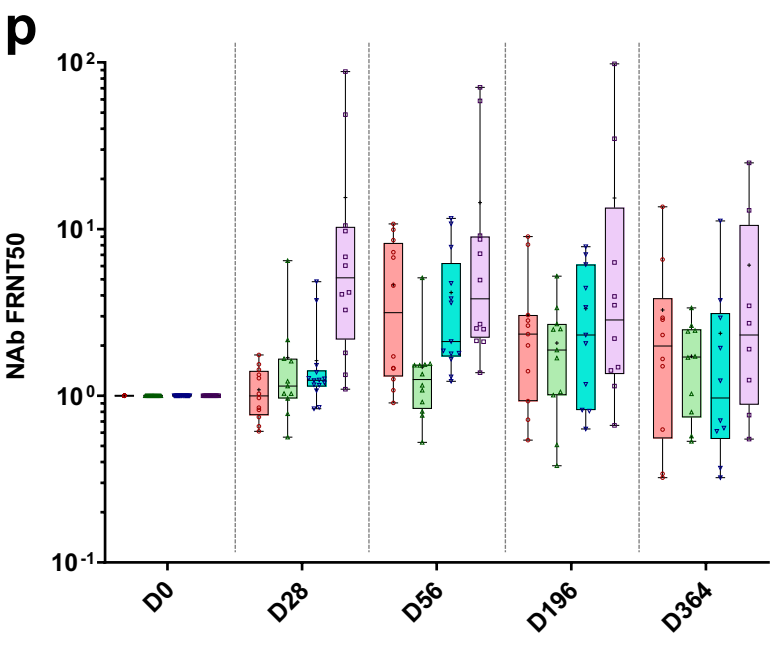

Supplementary Figure 7 Comparison of SARS-CoV-2 WT-specific humoral immune responses across vaccination groups at each time point

SARS-CoV-2 WT-specific humoral immune responses were compared between vaccination groups at each indicated time point. Each symbol represents an individual participant. Statistical significance was determined using one-way ANOVA with Tukey's multiple comparison test. P values are shown for group-wise comparisons within each time point (*P < 0.05; **P < 0.01; ***P < 0.001; ****P < 0.0001).

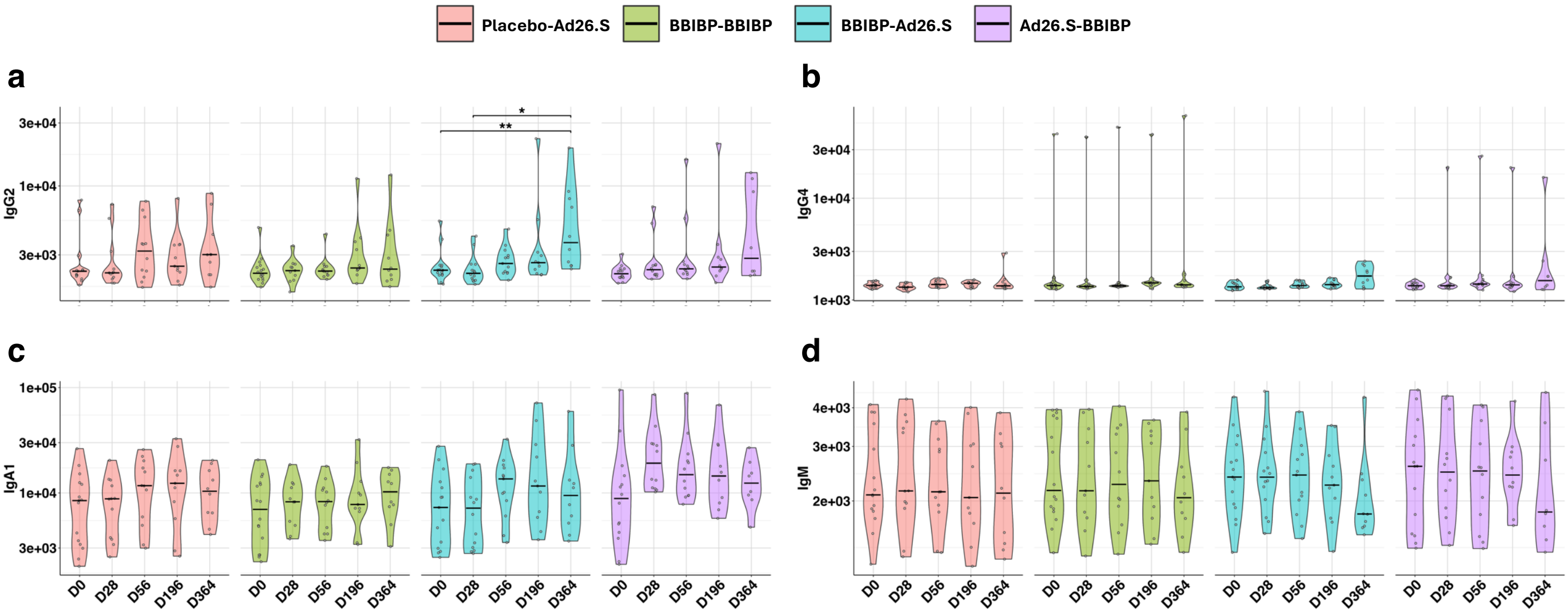

Supplementary Figure 8 SARS-CoV-2 Omicron BA.1 S-specific antibody isotypes and subclasses

Violin plots tracking the longitudinal **(a)** IgG2, **(b)** IgG4, **(c)** IgA1, and **(d)** IgM responses specific to SARS-CoV-2 Omicron BA.1 S protein are presented for the four vaccine groups over 364 days. Individual data points are shown as gray dots, with the median indicated by a black horizontal line. The y-axes represent the GMFI values for each antibody isotype and subclass. Assays were conducted in quadruplicate to ensure consistency. Antibody levels at different time points within each group were performed using Wilcoxon signed-rank test with Bonferroni correction (*P < 0.05, **P < 0.01, ***P < 0.001, and ****P < 0.0001). Statistical significance was determined exclusively for comparisons between D0 and D28.

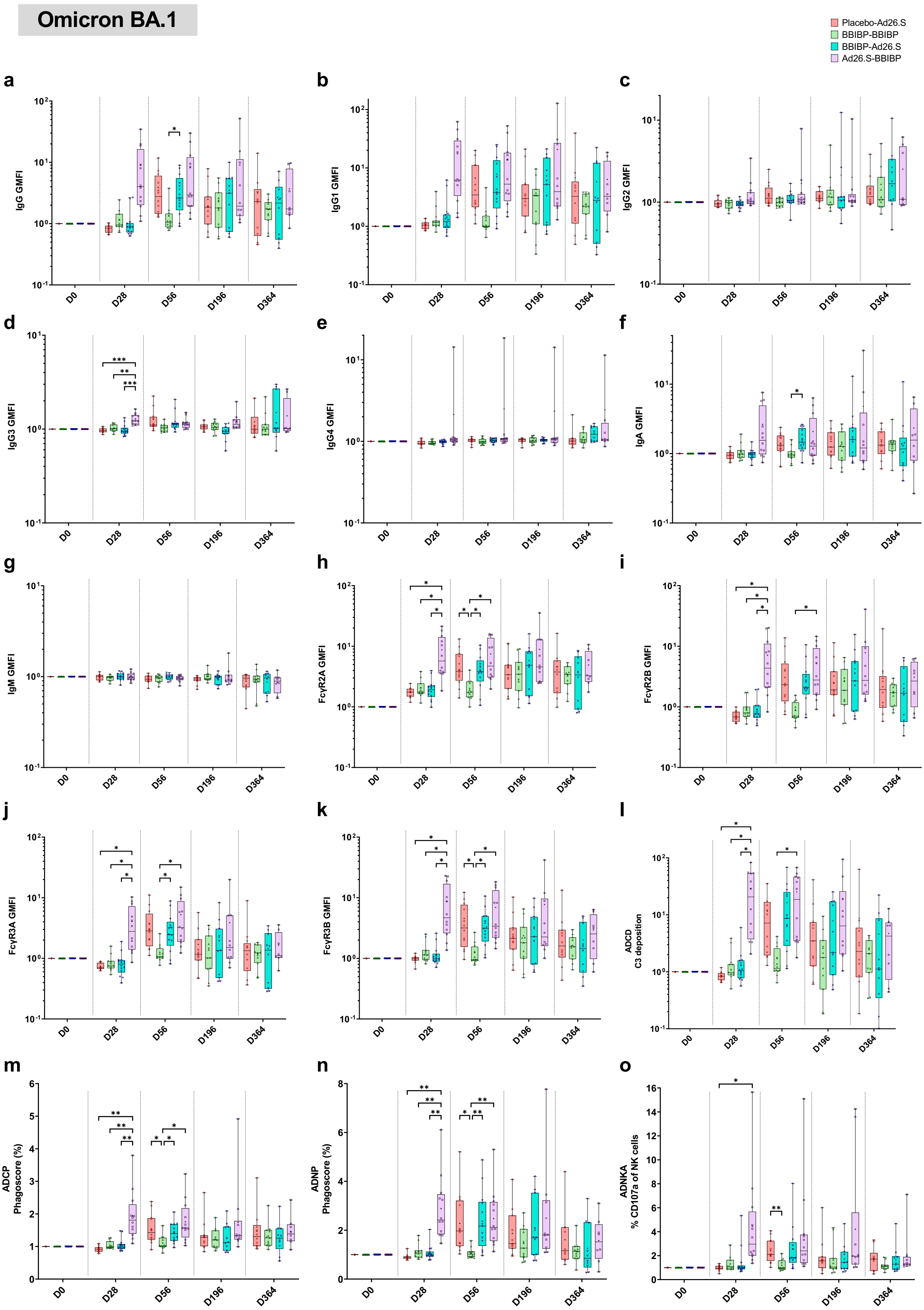

Supplementary Figure 9 Comparison of SARS-CoV-2 Omicron BA.1-specific humoral immune responses across vaccination groups at each time point

SARS-CoV-2 Omicron BA.1-specific humoral immune responses were compared between vaccination groups at each indicated time point. Each symbol represents an individual participant. Statistical significance was determined using one-way ANOVA with Tukey's multiple comparison test. P values are shown for group-wise comparisons within each time point (*P < 0.05; **P < 0.01; ***P < 0.001; ****P < 0.0001).

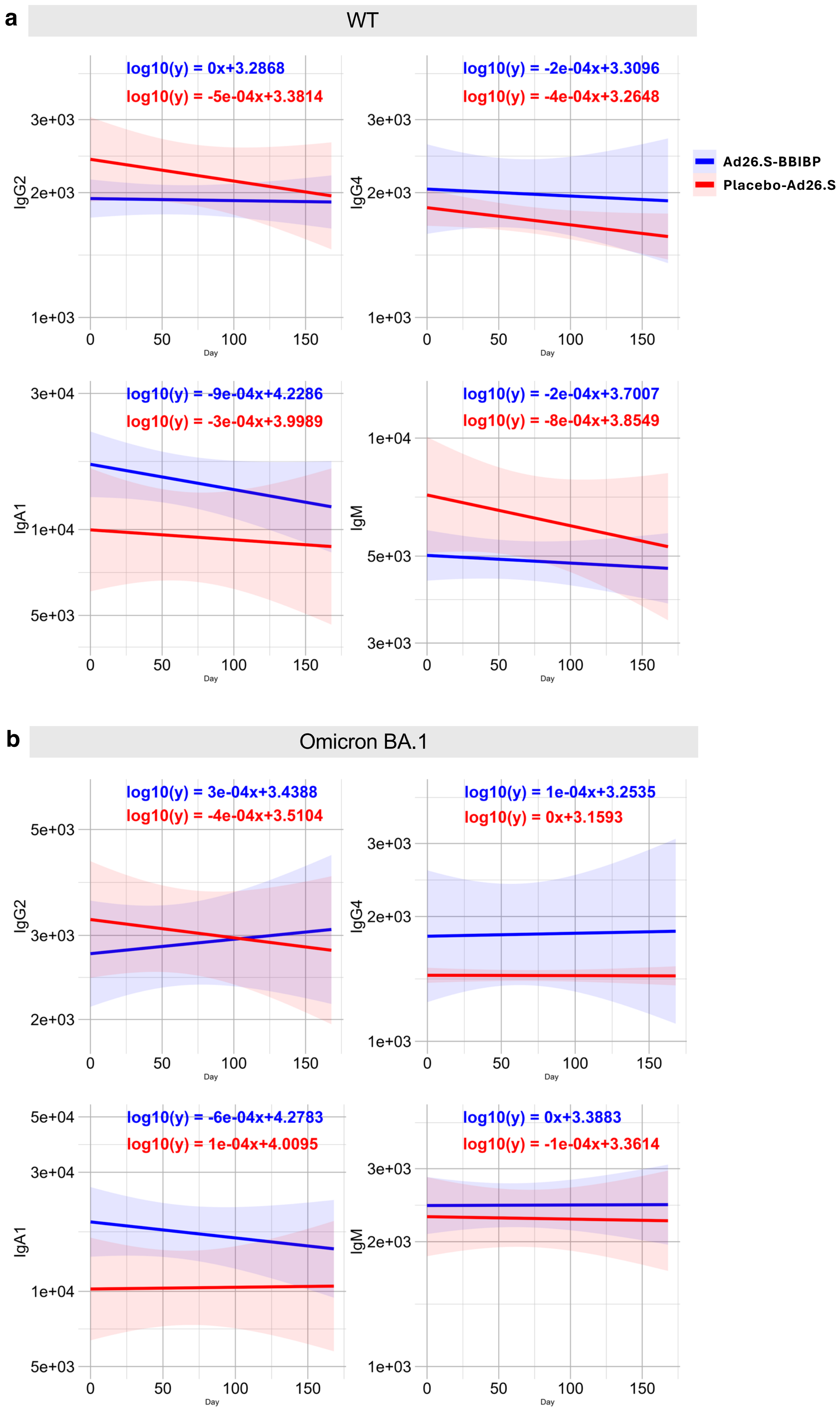

Supplementary Figure 10 Decay dynamics of antibody responses against SARS-CoV-2 WT and BA.1 S protein

Waning rates of IgG2, IgG4, IgA1, and IgM antibody levels against **(a)** WT and **(b)** BA.1 S proteins were evaluated over a 168-day period from peak response. Placebo-Ad26.S (red) and Ad26.S-BBIBP (purple) groups were compared using log-transformed GMFI values via linear regression modeling. Best-fit lines with 95% confidence intervals (shaded) are shown, and the equations are color-coded for each group.

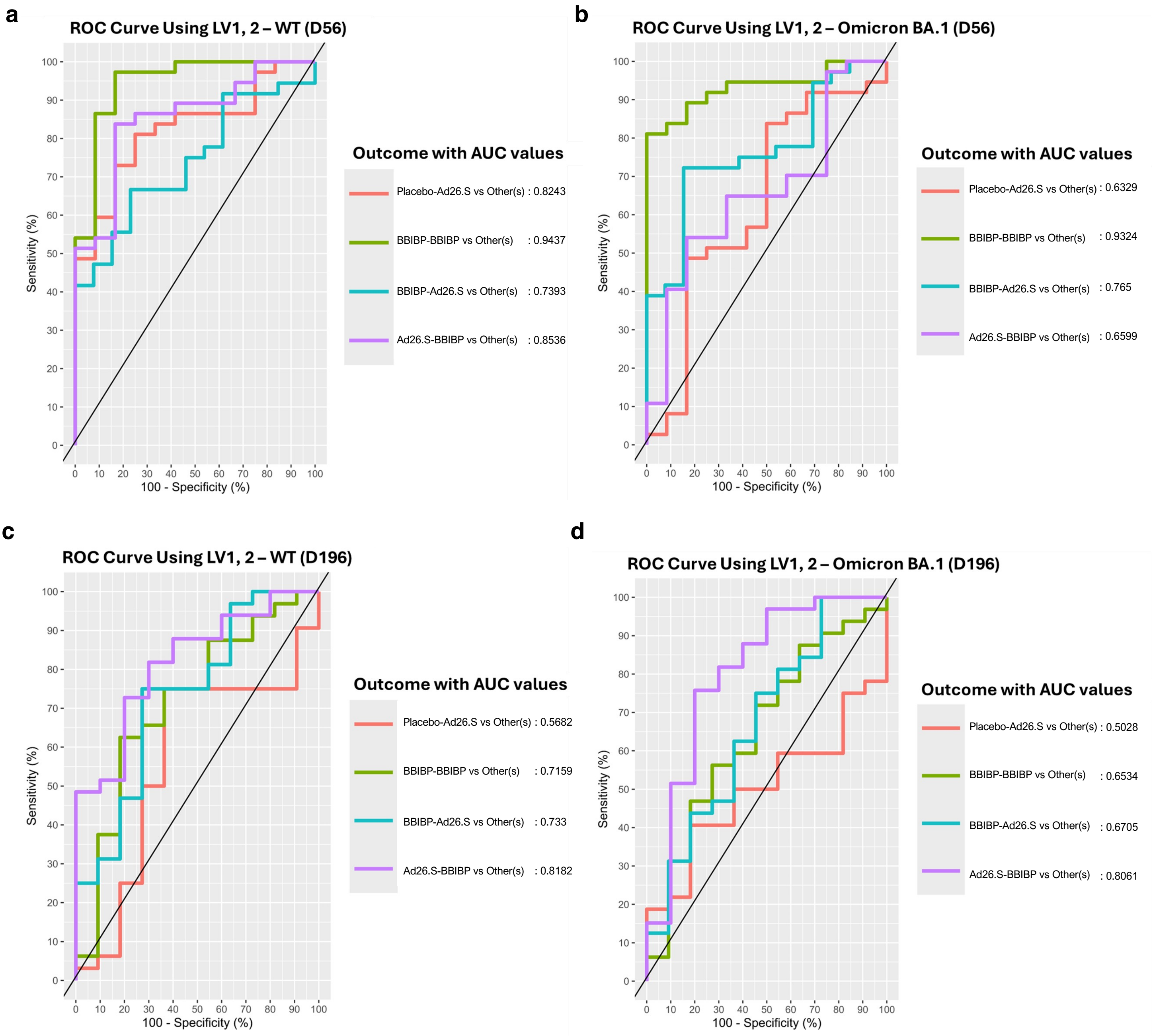

Supplementary Figure 11 Receiver operating characteristic (ROC) curve analysis of sPLS-DA model performance across vaccine regimens against SARS-CoV-2 WT and Omicron BA.1 at D56 and D196

ROC curves evaluating the discriminative performance of latent variables 1 and 2 (LV1 and LV2) derived from sPLS-DA models for classification of each vaccine regimen against others. Analyses were performed using humoral immune responses to SARS-CoV-2 WT S at **(a)** D56 and **(c)** D196, and Omicron BA.1 S at **(b)** D56 and **(d)** D196. Each curve represents a one-versus-rest comparison for a given vaccine regimen (Placebo–Ad26.S, red; BBIBP–BBIBP, green; BBIBP–Ad26.S, turquoise; Ad26.S–BBIBP, purple). The area under the curve (AUC) indicates the discriminative performance of the model.

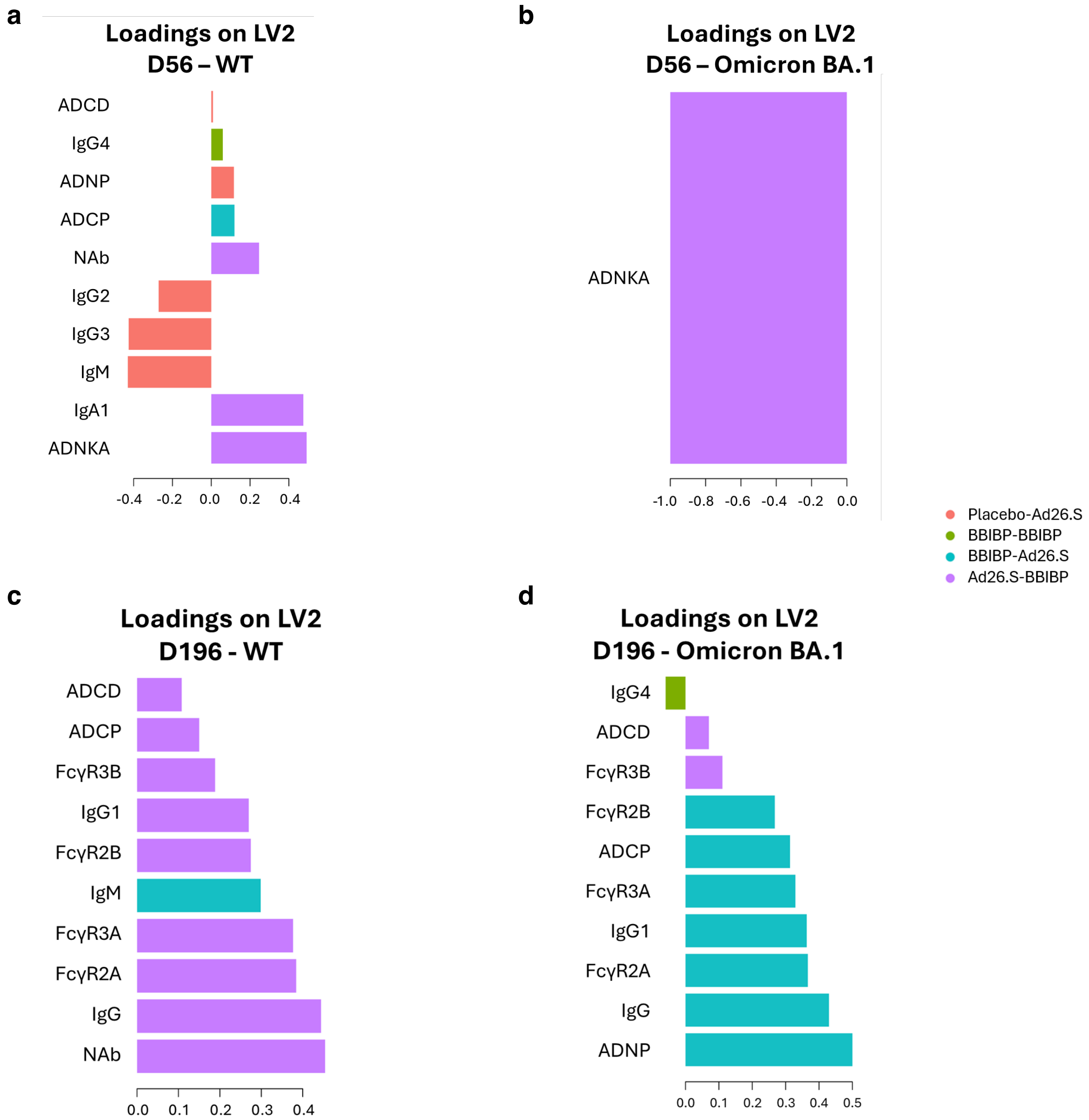

Supplementary Figure 12 Feature loadings on Latent Variable 2 (LV2) from sPLS-DA for SARS-CoV-2 WT and Omicron BA.1

Bar plots show the contribution (loading weights) of immune features to LV2 derived from sPLS-DA models using humoral responses to SARS-CoV-2 WT S at **(a)** D56 and **(c)** D196, and Omicron BA.1 S at **(b)** D56 and **(d)** D196. Bars represent the relative contribution of each feature to group separation along LV2, with higher absolute loading values indicating a stronger contribution. Colors indicate the vaccine regimens: Placebo-Ad26.S (red), BBIBP-BBIBP (green), BBIBP-Ad26.S (turquoise), and Ad26.S-BBIBP (purple).
